# Mixed Transcriptome Analysis Revealed the Possible Interaction Mechanisms between *Zizania latifolia* and *Ustilago esculenta* Inducing Jiaobai Stem-Gall Formation

**DOI:** 10.3390/ijms222212258

**Published:** 2021-11-12

**Authors:** Zhi-Ping Zhang, Si-Xiao Song, Yan-Cheng Liu, Xin-Rui Zhu, Yi-Feng Jiang, Ling-Tong Shi, Jie-Zeng Jiang, Min-Min Miao

**Affiliations:** 1 College of Horticulture and Plant Protection, Yangzhou University, Yangzhou 225009, China; zhangzp@yzu.edu.cn (Z.-P.Z.); ssx123606@163.com (S.-X.S.); lyc1234560214@sina.com (Y.-C.L.); zhuxr2001@163.com (X.-R.Z.); jyf11111019@163.com (Y.-F.J.); slt20010919@163.com (L.-T.S.); jzjiang@yzu.edu.cn (J.-Z.J.); 2 Jiangsu Key Laboratory of Crop Genomics and Molecular Breeding, Agricultural College, Yangzhou University, Yangzhou 225009, China

**Keywords:** *Zizania latifolia*, *Ustilago esculenta*, stem-gall formation, transcriptome, auxin, *YUCCA*

## Abstract

The smut fungus *Ustilago esculenta* infects *Zizania latifolia* and induces stem expansion to form a unique vegetable named Jiaobai. Although previous studies have demonstrated that hormonal control is essential for triggering stem swelling, the role of hormones synthesized by *Z. latifolia* and *U. esculenta* and the underlying molecular mechanism are not yet clear. To study the mechanism that triggers swollen stem formation, we analyzed the gene expression pattern of both interacting organisms during the initial trigger of culm gall formation, at which time the infective hyphae also propagated extensively and penetrated host stem cells. Transcriptional analysis indicated that abundant genes involving fungal pathogenicity and plant resistance were reprogrammed to maintain the subtle balance between the parasite and host. In addition, the expression of genes involved in auxin biosynthesis of *U. esculenta* obviously decreased during stem swelling, while a large number of genes related to the synthesis, metabolism and signal transduction of hormones of the host plant were stimulated and showed specific expression patterns, particularly, the expression of *ZlYUCCA9* (a flavin monooxygenase, the key enzyme in indole-3-acetic acid (IAA) biosynthesis pathway) increased significantly. Simultaneously, the content of IAA increased significantly, while the contents of cytokinin and gibberellin showed the opposite trend. We speculated that auxin produced by the host plant, rather than the fungus, triggers stem swelling. Furthermore, from the differently expressed genes, two candidate Cys2-His2 (C2H2) zinc finger proteins, GME3058_g and GME5963_g, were identified from *U. esculenta*, which may conduct fungus growth and infection at the initial stage of stem-gall formation.

## 1. Introduction

*Zizania latifolia* Turcz. is a perennial aquatic plant that is an ancient cereal crop and has been cultivated for more than 2000 years in China [1]. However, after being parasitized by *Ustilago esculenta*, *Z. latifolia* no longer undergoes inflorescence and forms seeds but stimulates the upper parts of the stem expansion and becomes a vegetable, which is a common occurrence in China, India, and Japan in Southeast Asia [2,3]. In nature, *U. esculenta* is a number of smut fungi belonging to the basidiomycetes. Smut fungi, such as *U. maydis*, *Sporisorium reilianum*, *U. hordei*, *Urocystis tritici*, and *Thecaphora solani*, consistently cause devastating effects on the host plant, preventing crop growth and causing huge economic losses; smut disease is sometimes the reason for famine in any particular area or place [4,5]. While during the long process of artificial selection and coevolution, *U. esculenta* and *Z. latifolia* reached a clever balance between plant’s disease resistance and fungal infection [3,6], the infected stem was no longer a common disease tumor (full of black powder) but formed an edible, white swollen culm gall, which was found to be delicious and nutritious, was deeply loved by the people and was called “Jiaobai” in China [6,7]. Jiaobai cultivation has become a pillar industry in some areas of the Zhejiang and Jiangsu provinces and other regions in China, and has high economic value [1,8].

For a long time, people have been trying to explore the unique mechanism underlying the formation of the fleshy stem of Jiaobai. *U. esculenta*, as a living trophic fungus, specifically infects *Z. latifolia* plants, Even in winter, smut fungi still exist in the old roots and stolon buds of the host plant. *U. esculenta* usually infects the buds of underground stems through vascular tissues, parenchyma or vascular bundles, after which linear, branching, aggregate, and polymerized hyphae and other changes along the intercellular space or through the cells of the host plant are observed during the transition from the seedling stage to fleshy, stem-swelling stage [3,8]. This process is also accompanied by a series of physiological changes, such as an increase in plant hormones, carbohydrate accumulation, and an enhancement of leaf photosynthesis during the expansion of stem galls. A large number of differentially expressed genes (DEGs) involved in material metabolism, signal transduction, plant–fungal interactions and other pathways have been found in transcriptome and proteome analyses during the different developmental stages of *Z. latifolia* [3,9,10].

Auxin plays a variety of roles in plant growth and development. Indole-3-acetic acid (IAA), the most common auxin, not only regulates cell division, elongation and differentiation and induces the formation of roots, stems, leaves, flowers and fruits, but it also participates in tropism, such as gravitropism and phototropism, and plays an important role in emergency responses and interactions with microorganisms [11,12,13,14]. Because many pathogens produce auxin during their interaction with plants, this hormone has been thought to be important in plant disease development [15,16]. The role of plant hormones, especially IAA, in stem-gall expansion of *Z. latifolia* has long been a concern of researchers [9,10,17]. However, both *Z. latifolia* and *U. esculenta* have the ability to synthesize IAA, and the chemical structures of IAA synthesized by plants and *U. esculenta* are identical and cannot be distinguished by labeling under symbiotic conditions. Thus, in the plant–fungus symbiotic system, which species is responsible for the synthesis of the auxin that triggers culm gall expansion remains unclear. In recent years, studies on *U. maydis* and other fungi have shown that IAA is mainly synthesized through the IPyA (indole-3-pyruvic acid) and IAM (indole-3-acetamide) pathways [18,19]. The key enzymes in the IPyA pathway include tryptophan aminotransferase (TAM), indole-3-pyruvate decarboxylase (IPDC) and indole-3-acetaldehyde dehydrogenase (IAD); the IAM pathway includes L-amino acid oxidase (iaaM) and N-acetylthanolamine amidohydrolase (iaaH) [20,21]. On the other hand, the tryptophan-dependent auxin biosynthesis pathway is essential for plant developmental processes, and TRYPTOPHAN AMINOTRANSFERASE OF ARABIDOPSIS (TAA) and *YUCCA* (a flavin monooxygenase) are two important catalytic enzymes [22,23]. The difference in the IAA biosynthesis pathway between *U. esculenta* and plants could provide the possibility of distinguishing the producer of IAA in the initial formation of Jiaobai stem gall. Moreover, the *YUCCA* protein is the rate-limiting enzyme in the Trp-dependent IAA biosynthesis pathway, and the *YUCCA* gene has been studied extensively in many plants, such as *Arabidopsis*, maize (*Zea mays*), potato (*Solanum tuberosum*), and rice (*Oryza sativa*), by identification and analysis at the whole-genome level [24,25,26,27]. Compared with the above-mentioned plants, our understanding of the *YUCCA* family in *Z. latifolia* is still limited.

To comprehensively understand plant–pathogen interactions, it is valuable to monitor the gene expression profiles of both interacting organisms simultaneously in the same infected plant tissue using the transcriptome [28,29,30]. This approach was used to investigate the DEGs in a variety of host plants and pathogens, such as in wheat defense and *Azospirillum brasilense* attack, the interaction between rice and *Magnaporthe oryzae*, and *Brassica napus* and *Sclerotinia sclerotiorum* [29,31,32]. To better understand the potential molecular mechanisms of *Z. latifolia*–*U. esculenta* interactions and the role of plant hormones during the formation of stem galls, we performed dual (plant and fungus) RNA-seq transcriptional profiling of the DEGs of both *Z. latifolia* and *U. esculenta* and investigated the contents of phytohormones in the initial stage of stem-gall formation. We further identified the *YUCCA* gene family in the host using bioinformatics methods and analyzed the expression pattern of *YUCCA* gene family members when triggering the formation of stem galls. In addition, the pathogenicity genes in *U. esculenta* and plant-resistance genes in *Z. latifolia*, and *U. esculenta* differentially expressed transcripts factors were also analyzed to understand the role of both the plant and the fungus in the gall formation.

## 2. Results

### 2.1. Distribution of U. esculenta in the Initial Trigger of Stem-Gall Formation of Z. latifolia

**Figure 1:**
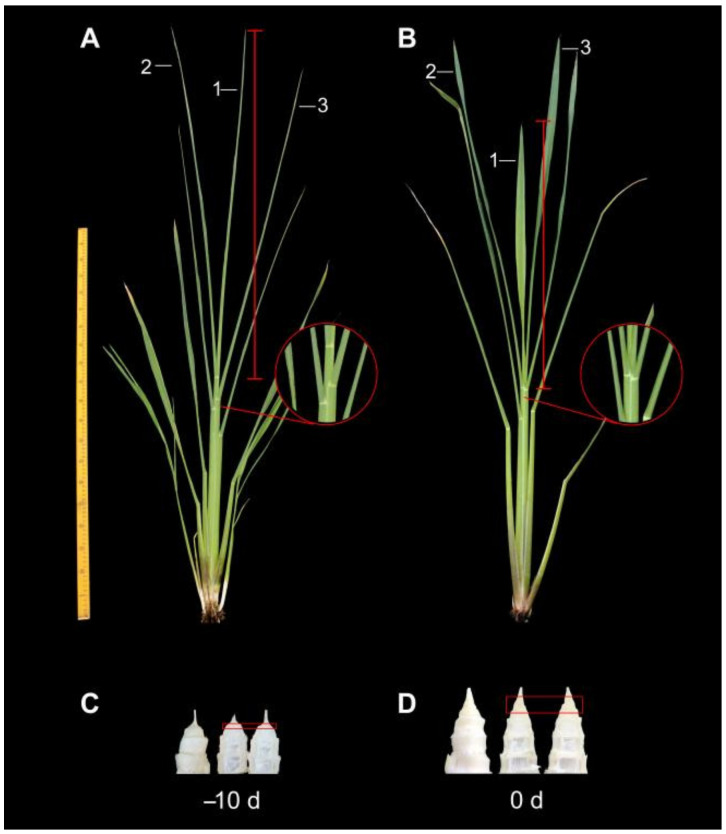
Photographs showing the morphology of plants and stem galls of Jiaobai. (**A**,**B**) External morphological characteristics of Jiaobai plants. (**C**,**D**) Longitudinal section of stem gall. −10 d, 10 days before stem-gall formation; 0 d, the initial stage of stem-gall formation; red circles, the junction of leaf sheath and leaf. Red frame, the tissue for the mixed transcriptome analysis of *Z. latifolia* and *U. esculenta*.

At 10 days before culm gall formation (−10 d), the length of the three newly grown leaves (leaf 1, leaf 2 and leaf 3) were almost the same, and the junctions of the leaf sheath and leaf 1, leaf 2 and leaf 3 were obviously separated (Figure 1A, marked by red circles); when the outer leaves were stripped, the tops of the stem were only a very small and short growth point, and no obvious nodes were observed (Figure 1C). Upon the initial trigger of culm gall formation (0 d), the height of the central leaf (leaf 1) was lower than that of the outer leaf (leaf 2 and leaf 3), and its width became wider and the color became light green; the junctions of the leaf and the leaf sheath of leaves 1–3 gradually approached to form a line, and the nodes and internodes could be clearly observed (Figure 1B,D). These morphological changes provided useful cues for us to sample the stem apex at certain growth stages.

**Figure 2:**
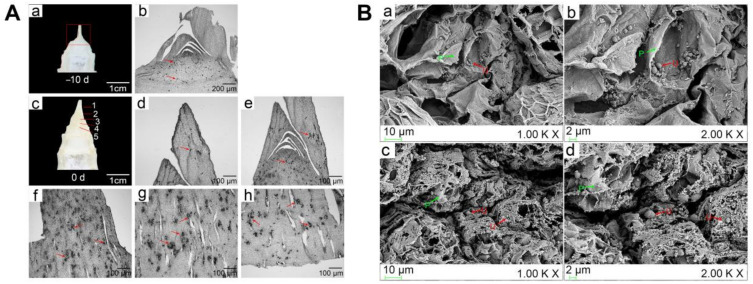
Observation of *U. esculenta* hyphal distribution in *Z. latifolia* tissue. (**A**) Paraffin sections showing the *U. esculenta* hyphal distribution in *Z. latifolia* stem apexes. (**a**,**b**). Longitudinal sections of the stem apex (**a**) and *U. esculenta* hyphal distribution (**b**) at −10 d before the stem began swelling (−10 d), bar = 200 μm. (**c**). Five tissue parts of the initial swollen stem tissue (0 d). (**d**–**h**). Distribution of *U. esculenta* hyphae. 1, (**d**) stem apex, 2, (**e**) first node, 3, (**f**) second node, 4, (**g**) internode, and 5, (**h**) bottom, respectively. bar = 100 μm. red arrows, *U. esculenta*. (**B**) Scanning electron micrographs of *U. esculenta* hyphae in *Z. latifolia* tissue. (**a**,**b**) −10 d before stem-gall formation. (**c**,**d**) 0 d of the initial stage of stem-gall formation. (**a**,**c**) and (**b**,**d**) bar = 10 μm and 2 μm. Red and green arrows represent *U. esculenta* and *Z. latifolia* tissue, respectively. Green letters P, *Z. latifolia*; red letters U, *U. esculenta*.

*U. esculenta* hyphal distribution at −10 d and 0d were observed. *U. esculenta* hyphae were rare in the −10 d and were focused at the nodes; most hyphae were short and rod-shaped, and a few were elongated (Figure 2A(a,b)). The initial swollen stem gall was divided into five parts: stem apex, first node, second node, internode, and bottom (Figure 2A(c)). The distribution of hyphae in the stem apex was small and compact, with a few hyphae (Figure 2A(d)). Below the apex, the number of hyphae began to increase significantly, and most of them were clustered at the first node of the stem gall (Figure 2A(e)). The number of small hyphal clusters in the second node was large and dense (Figure 2A(f)). The internode cells began to grow and gradually formed cavities, the area of small hyphal clusters in this region became larger, and the hyphae extended through a number of cells (Figure 2A(g)). At the bottom of the gall, the number and areas of hyphal clusters decreased, and the cavities became increasingly larger (Figure 2A(h)). Under scanning electron microscopy, a small number of round or irregular spores were sporadically distributed in the nonswollen tissue, while a large number of hyphae were dense and vigorous, showed a network and winding pattern, and tended to extend to the interior of the host tissue at 0 d of stem-gall formation (Figure 2B).

### 2.2. General Features of the U. esculenta and Z. latifolia Transcriptomes

**Figure 3:**
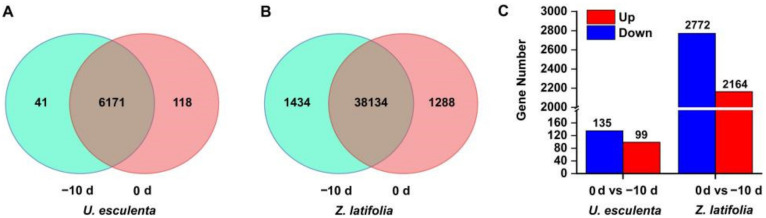
Transcriptome profiles of the gene expression patterns of *Z. latifolia* and *U. esculenta* in the initial stage of stem-gall formation. The number of common genes, specific genes of *U. esculenta* (**A**) and *Z. latifolia* (**B**), and DEGs in both interacting organisms (**C**) between −10 d and 0 d of stem-gall formation.

To identify the genes expressed in fungi and plants upon the initial trigger of culm gall formation of *Z. latifolia*, we selected the early stage of swollen stem, i.e., 0 d of culm gall formation (0 d), and used 10 d before culm gall formation (−10 d) as the control. In this project, a total of six samples were tested using the BGISEq-500 platform, and each sample produced 15.25 Gb of clean bases on average. The first versions of the *Z. latifolia* genome database (ASSH00000000.1) and *U. esculenta* genome database (JAAKGJ010000000) were selected as reference sequences. The average ratio of the samples to the *Z. latifolia* and *U. esculenta* genomes was 84.03% and 4.76%, and the average ratio of the gene set was 66.82% and 3.56%, respectively (Table 1). The correlation coefficient analysis of the samples showed a clear distinction between the transcriptomes of −10 d before and 0 d of stem-gall formation (Figure S1). The gene mapping to *Z. latifolia* showed a greater abundance at −10 d than at 0 d (the total mapping of the gene set was 67.5% at −10 d and 66.1% at 0 d), while the results from *U. esculenta* showed the opposite pattern. The total mapping of the gene set at 0 d was 1.47-times greater than that at −10 d (Table S1 and Figure 3).

A total of 6330 *U. esculenta* genes were present in the interacting transcriptome, among which 6319 were known and 11 were novel. A total of 6171 occurred at both 10 d and 0 d, among which 41 genes were known in the −10 d samples and 118 genes were known in the 0 d samples (Figure 3A). Furthermore, 234 DEGs were identified upon *Z. latifolia* enlargement, and 99 genes were upregulated and 135 genes were downregulated in the 0 d compared with the −10 d samples (Figure 3C).

When the *Z. latifolia* genome was used as a reference, the total number of expressed genes was 40,856, including 37,622 known genes and 3234 predicted new genes; a total of 36,704 new transcripts were detected, among which 24,993 belonged to new alternative splicing subtypes of known protein-coding genes, 3280 belonged to transcripts of new protein-coding genes, and the remaining 8431 belonged to long noncoding RNA (Table 1). There were 38,134 common genes in both samples and 1434 and 1288 unique genes at −10 d and 0 d, respectively (Figure 3B). Compared with the −10 d samples, there were 4936 DEGs, 2164 upregulated genes and 2772 downregulated genes in the 0 d samples (Figure 3C). Further analysis of the Gene Ontology (GO) and the KEGG pathway of DEGs in *U. esculenta* and *Z. latifolia* can be seen in the Supplementary Materials (Figures S2–S5, and Tables S2–S6).

### 2.3. Analysis of Pathogenicity Genes in U. esculenta and Plant Resistance Genes in Z. latifolia

**Figure 4:**
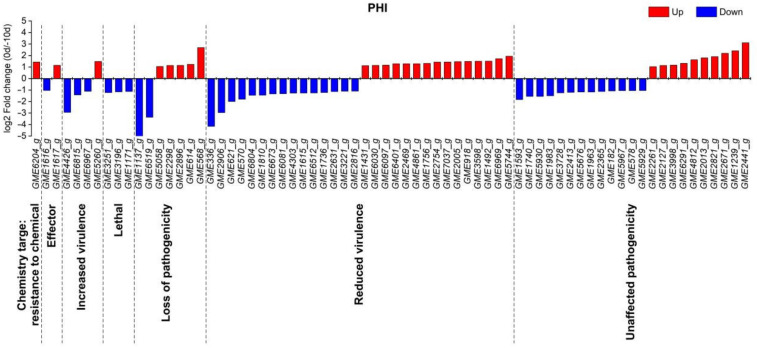
Prediction of pathogenicity-related genes based on the PHI database. Chemical target: resistance to a chemical, in which the mutation of the gene leads to resistance to certain drugs; Effector (plant avirulence determinant); Increased virulence, Hypervirulence; Lethal, the gene is necessary for the survival of the strain; Loss of pathogenicity, deletions of the gene do not cause disease; Reduced virulence, the deletion mutant is less virulent; Unaffected pathogenicity, the gene does not affect the pathogenicity of the pathogen.

The PHI (pathogen-host interactions) database integrates a large amount of experimental gene data for known virulence, lethality and effector functions of pathogens infecting animals, plants and fungi. In this study, we used DIAMOND v0.8.31 (Max Planck Institute for Developmental Biology, Tübingen, Germany) to align genes to the PHI base database for annotation. Using a query coverage ≥50% and identity ≥40% as filter parameters, a total of 71 DEGs demonstrated homology with known pathogenicity genes. Among them, 44 DEGs were considered to be predictive pathogenic factors of *U. eculenta*, including 4 DEGs for increased virulence, 7 DEGs for lost pathogenicity, 30 DEGs for reduced virulence, and 2 DEGs for effectors. Concurrently, 3 DEGs and 1 DEG were found to be homologous with the PHI genes having lethal properties and targeted resistance to chemicals, respectively. It is worth noting that the expression levels of three DEGs, *GME3251_g*, *GME3196_g* and *GME1171_g*, which were necessary for the survival of the strain, were all significantly lower in the initial stage of culm gall formation than at 10 d before gall enlargement. In addition, most of the DEGs with increased virulence such as *GME4426_g*, *GME6815_g* and *GME6967_g*, also showed the same expression pattern (Figure 4).

Plant resistance genes play an important role in the interaction between plants and pathogens. There were 368 DEGs in the initial stage of stem-gall formation, including 31 DEGs in the combination of coiled coil domain, nucleotide binding site and leucine-rich repeat (CC-NB-LRR, or CNL), 104 DEGs in the combination of nucleotide binding site and leucine-rich repeat (NB-LRR, or NL), 28 DEGs in the combination of Toll-interleukin receptor-like domain, nucleotide binding site and leucine-rich repeat (TIR-NB-LRR, or TNL), 73 DEGs in the combination of receptor serine–threonine kinase-like domain and extracellular leucine-rich repeat (ser/thr-LRR, or RLP), and 22 DEGs in the combination of kinase domain and extracellular leucine-rich repeat (Kin-LRR, or RLK), which represented the domain types of plant disease-resistance genes with more DEGs (Figure S6).

### 2.4. Hormonal Analysis during Stem-Gall Formation in Z. latifolia

#### 2.4.1. Hormone Content Analysis

**Figure 5:**
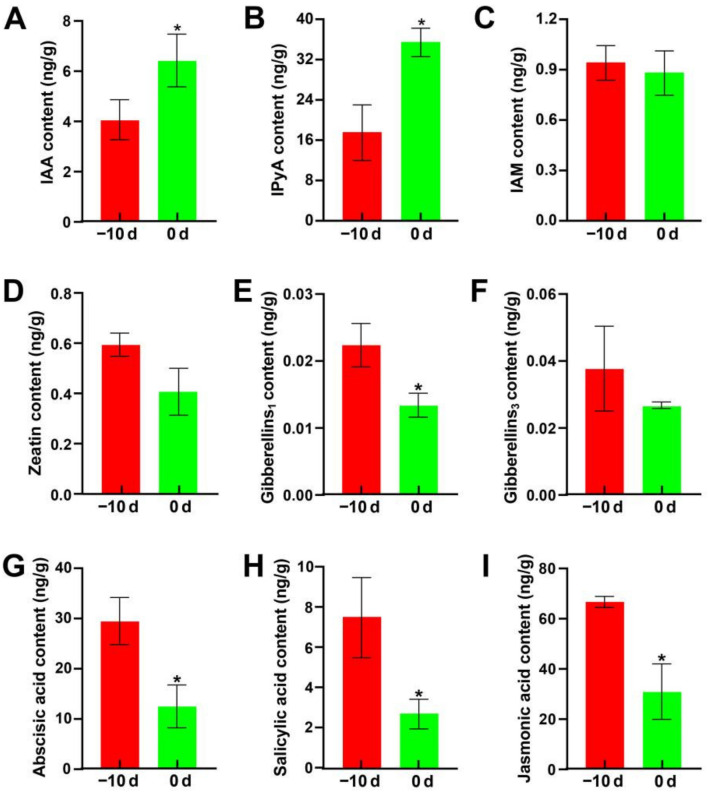
Hormone content at the early stage of stem-gall formation. (**A**–**I**) IAA, IPyA, IAM, ZT, GA_1_, GA_3_, ABA, SA and JA. Asterisks (*) indicate significant differences (*p* < 0.05) between same form of hormone content in the different stage of stem-gall formation.

The role of phytohormones in the gall emlargement of “Jiaobai” has been widely reported. Therefore, analysis of hormone content at the early stage of stem-gall formation was conducted. The hormone content assay in the apical stem meristem indicated that the free indole-3-acetic acid (IAA) content and IPyA content increased significantly at 0d compared with −10 d, while the contents of six other hormones, zeatin (ZT), GA_1_, GA_3_, ABA, JA and SA, decreased by 31.5%, 40.3%, 29.6%, 57.5%, 64.1% and 53.7%, respectively (Figure 5). These results further indicate that IAA should play a key role in the induction of fleshy stem enlargement in *Z. latifolia.*

#### 2.4.2. DEGs Involved in Hormone Metabolism and Signal Transduction

Furthermore, all the DEGs involved in hormone metabolism and signal transduction of *U. esculenta* and *Z. latifolia* during stem-gall formation were also investigated. Tryptophan is an essential precursor of many indoles and other secondary metabolites, and anthranilate synthase (TrpE) is a key regulatory enzyme in the synthesis of tryptophan, indole-3-acetic acid, and indole alkaloids. In *U. esculenta*, we found that the expression of anthranilate synthase encoded by *GME3221_g* (annotated in the KEGG pathway: phenylalanine, tyrosine and tryptophan biosynthesis) decreased significantly. Furthermore, expression of the genes encoding enzymes involved in auxin synthesis (annotated in the KEGG pathway: tryptophan metabolism), such as *GME1810_g* encoding tryptophan aminotransferase and *GME621_g* and *GME6394_g* encoding aldehyde dehydrogenases, was significantly decreased in the initial stage of culm gall formation. This result suggested that auxin synthesis in fungi was less efficient during the formation of *Z. latifolia* (Table S4).

**Figure 6:**
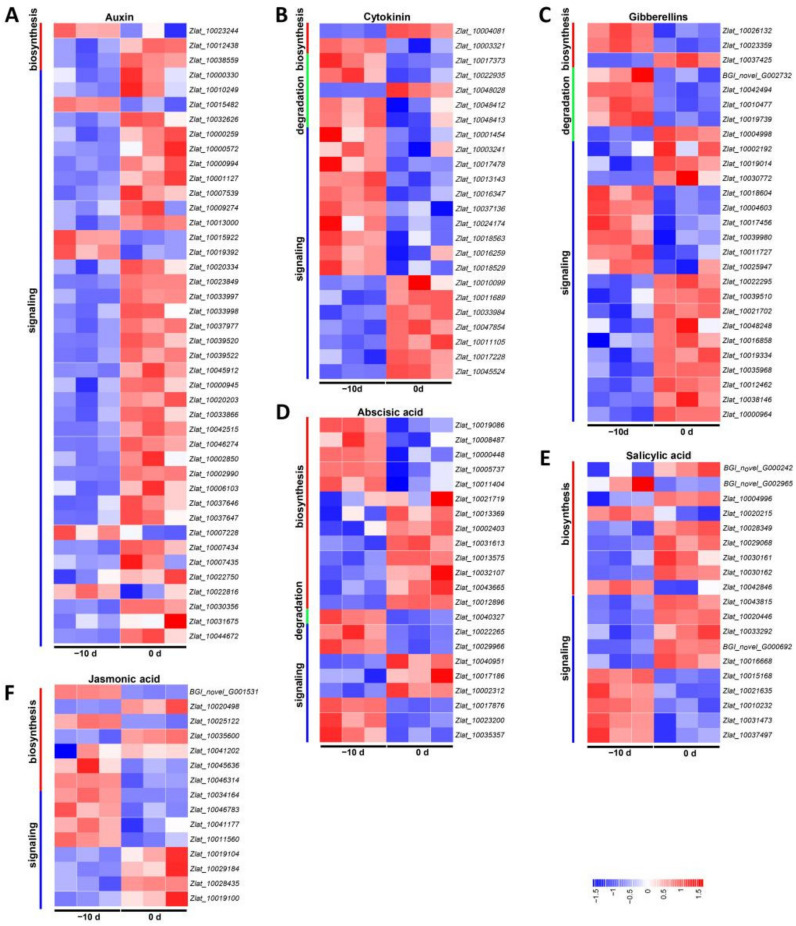
Expression profiles of DEGs related to hormone metabolism and signals between the two stages (−10 d and 0 d) of stem-gall formation based on the *Z. latifolia* genome in RNA-Seq. (**A**–**F**) DEGs for auxin, cytokinin, gibberellin, abscisic acid, salicylic acid and jasmonic acid. Red, green and blue lines represent biosynthetic degradation and signal transduction DEGs, respectively.

In *Z. latifolia*, the results revealed 149 DEGs associated with the auxin (IAA), cytokinin (CTK), gibberellin (GA), abscisic acid (ABA), salicylic acid (SA) and jasmonic acid (JA) pathways (Figure 6 and Table S7). Among the six hormones, the auxin synthesis and signal transduction pathways had the largest number of DEGs, where the expression of 36 of the 43 differential genes was upregulated, and only 6 genes were downregulated, indicating auxin should play a pivotal role in the gall formation. In the cytokinin group, however, most DEGs were downregulated, accounting for 62.5%. The other DEGs were related to gibberellin, abscisic acid and salicylic acid and jasmonic acid metabolism and signal transduction pathways, which encompassed 15, 11, 11 and 7 upregulated DEGs and 12, 11, 8 and 8 downregulated DEGs, respectively. Among 149 DEGs, the most upregulated and downregulated genes were *Zlat_10044672* (auxin-induced protein, 15A-like) and *Zlat_10015168* (a putative bZIP transcription factor in the salicylic acid pathway), respectively (Table S7). Notably, according to the sequence analysis, among the three genes annoatated in the auxin biosynthesis pathway, the only downregulated gene (*Zlat_10023244*, annoated as cytochrome P450 77A3) does not belong to the *YUCCA* family, while the expression level of another typical *YUCCA* gene *Zlat_100038559* was significantly increased. These results further suggest that auxin was important for gall formation. The annotation and the Log2-fold changes of involved genes are listed in Table S7.

### 2.5. Auxin Regulation of Stem-Gall Formation in Z. latifolia

#### 2.5.1. Genes Encoding Enzymes Involved in IAA Biosynthesis in *U. esculenta* and *Z. latifolia*

Using BLASTP, we obtained 2 TAM (GME1810_g, GME6257_g), 1 IPDC (*GME2840_g*), 4 IAD (GME621_g, GME4730_g, GME6394_g, GME6395_g), 1 IaaM (*GME5774_g*), 2 iaaH (GME5840_g, GME6749_g) and 2 Nitrilase (GME1046_g, GME2826_g) from *U. esculenta* and 4 TAA (Zlat_10020352, Zlat_10036150, Zlat_10029220, Zlat_10033167) and 1 IAMH (Zlat_10032813) from *Z. latifolia* (Table S8). The YUCCA contained the conserved domains of FMO (FxGxxxHxxxY), FAD (GxGxG) and NADPH binding (GxGxG). Therefore, to identify the *YUCCA* gene family of *Z. latifolia*, we not only used the *Arabidopsis YUCCA* protein sequence for BLASTP but also analyzed the basic domain. In addition, because of the unusual FMO-identifying motifs (FxSxxxHxxxY) in *Arabidopsis* YUCCA1, we also identified these unusual motifs in all *Z. latifolia* genes. Finally, a total of 12 genes, *Zlat_10027383*, *Zlat_10004518*, *Zlat_10004101*, *Zlat_10029766*, *Zlat_10013524*, *Zlat_10029560*, *Zlat_10017358*, *Zlat_10020875*, *Zlat_10038559*, *Zlat_10020360*, *Zlat_10002766*, *and Zlat_10020876*, were identified as the *Z. latifolia YUCCA* gene and named *ZLYUCCA1-12* (Tables S8 and S9).

#### 2.5.2. Structural and Functional Characteristic Analysis of the YUCCA Gene Family in *Z. latifolia*

**Figure 7:**
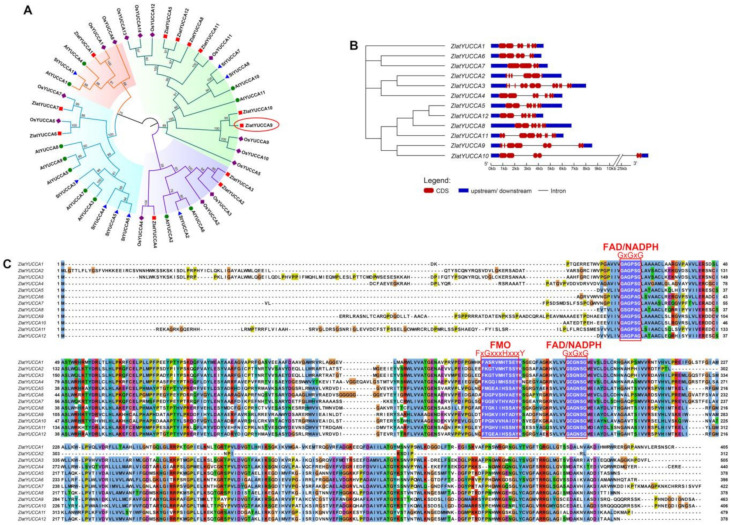
*YUCCA* gene family analysis. (**A**) Phylogenetic analysis of the YUCCA protein in *Z. latifolia*, *Arabidopsis*, rice and potato. (**B**) Structural analysis of the YUCCA family. (**C**) The complete protein sequences of YUCCA family proteins were aligned by ClustalW. The FMO (FxGxxxHxxxY), FAD (GxGxG) and NADPH-binding (GxGxG) domains are marked with a red box.

The 11, 14 and 8 YUCCA protein sequences from *Arabidopsis*, rice and potato were used as references to construct a phylogenetic tree with 12 YUCCA protein sequences of *Z. latifolia* plants in order to explore the phylogenetic relationship of the *YUCCA* gene family in *Z. latifolia* (Figure 7A, Table S9). The results showed that the *Z. latifolia YUCCA* gene family was mainly divided into four subfamilies, *ZlYUCCA 1*, *ZlYUCCA* *2,3,4, ZlYUCCA 6,7* and *ZlYUCCA 5, 8, 9, 10, 11, 12*. In addition, the phylogenetic tree showed that *Z. latifolia* was closely related to rice but distantly related to *Arabidopsis* and potato. Structural analysis of the *YUCCA* gene family in *Z. latifolia* showed that the gene structure was complex, and the number of exons and introns was irregular, with 2–8 and 1–7, respectively (Figure 7B). Furthermore, the complete protein sequences of YUCCA family proteins in *Z. latifolia* were aligned, and the three conserved domains FMO (FxGxxxHxxxY), FAD (GxGxG) and NADPH binding (GxGxG), were labeled. Among the 12 YUCCAs, ZlYUCCA1 (Zlat_10027383) contained a nonstandard FMO motif, the G mutation in FXGXXXHXXXY was S, and the similarity between the amino acid sequence of this gene and OsYUCCA1 was 93.675% (Figure 7A,C).

#### 2.5.3. Gene Expression of Enzymes Related to IAA Biosynthesis at the Early Stage of Stem-Gall Formation by qRT-PCR

**Figure 8:**
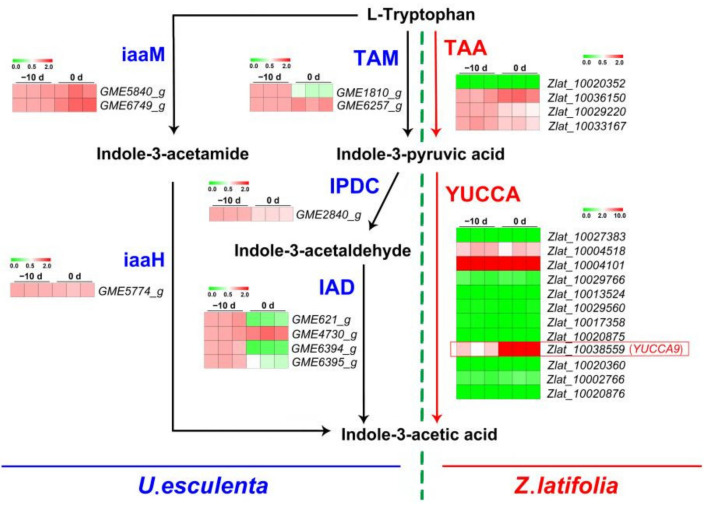
Validation of the RNA-Seq data of key enzymes in the IAA biosynthesis pathway using qRT-PCR at the initial stage of stem-gall formation. Black lines and blue letters denote the IAM pathway and IPyA pathway and the key enzyme in IAA synthesis in *U. esculenta*. Red lines and letters indicate the IAA synthesis pathway and key enzymes in the host plant *Z. latifolia*. The relative expression was determined by the 2^−ΔΔCT^ method. MeV version 4.9.0 software (Dana-Farber Cancer Institute, Boston, MA, USA) was used for the statistical analysis.

To elucidate the sources of auxin biosynthesis, the expression of genes related to IAA biosynthesis in *Z. latifolia* and *U. esculenta* in the initial stage of culm gall formation was analyzed (Figure 8, Table S10). Compared with −10 d, the expression levels of 1 TAM (*GME6257_g*) and 2 IADs (*GME621_g* and *GME6394_g*) were significantly decreased, and there was no significant difference in the expression of other enzyme genes during IAA synthesis of *U. esculenta* between two development stages. In *Z. latifolia*, the change in TAA family genes was not obvious, but particularly interestingly, in the *YUCCA* family, *ZlYUCCA9* (*Zlat_10038559*) was significantly upregulated in the initial stage of culm gall formation. The expression pattern of other genes involved in IAA biosynthesis pathways is listed in Table S10, and no remarkable expression level difference of these genes was found. The qRT-PCR results were similar to those of RNA-Seq, which showed a consistent expression trend and indicated that the DEGs obtained by RNA-Seq were reliable. These results also implied that the auxin inducing the initial expansion of the culm gall was mainly biosynthesized by the host plant *Z. Latifolia* rather than the fungus *U. esculenta*, and *ZlYUCCA9* played a key role in this process.

### 2.6. Expression Pattern of Cys2–His2 (C2H2) Zinc Finger Proteins of U. esculenta at the Early Stage of Stem-Gall Formation

**Figure 9:**
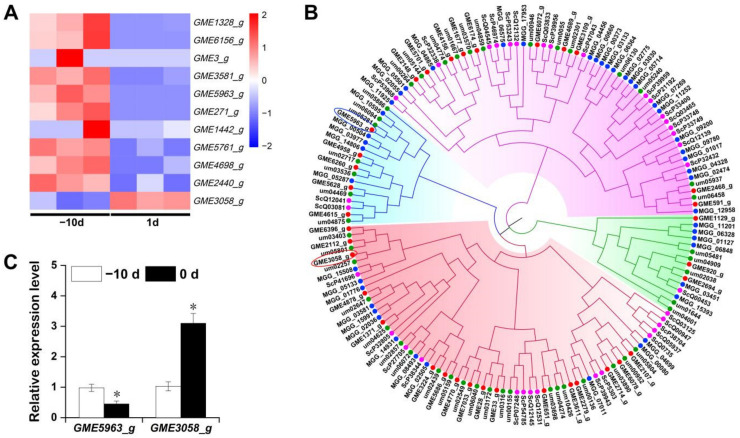
Analysis of C2H2 zinc finger proteins of *U. esculenta*. (**A**) Heatmap analysis of C2H2 DEGs in RNA-Seq at the early stage of stem-gall formation. (**B**) Phylogenetic analysis of the C2H2 zinc finger proteins in *U. esculenta* (red), *U. maydis* (green), *M. oryzae* (blue) and *S. cerevisiae* (amaranth). red circle: GME3058, blue circle: GME5963_g. (**C**) Validation of *GME3058_g* and *GME5963_g* expression levels in RNA-Seq data using RT-PCR. Asterisks (*) indicate significant differences (*p* < 0.05) between same gene in the different stage of stem-gall formation.

To investigate how *U. esculenta* participate in the biological process of gall initiation, differently expressed transcript factors (TFs) of the fungus during gall formation were identified. Interestingly, all 11 differently expressed transcripts annotated as TFs during this stage belong to the *C2H2* family, indicating that these zinc finger proteins may play key roles of Jiaobai gall formation (Figure 9A). Further sequence analysis suggests that all 10 downregulated *C2H2* transcripts have high sequence similiarity and may be produced by alternative splicing from a single gene (Figure S7). Thus, the longest transcript *GME5963_g* was used as a representative for further analysis. Using BLASTP, 35 *C2H2* family genes were obtained from the *U. esculenta* genome (Table S11). The structure of *C2H2* family genes was simple, and the number of introns is no more than 4, of which 18 genes have no introns (Figure S8). Therefore, 35, 46, 45 and 35 C2H2 protein sequences from *U. esculenta*, *U. maydis*, *M. oryzae* and *Saccharomyces cerevisiae* were collected for phylogenetic analysis (Figure 9B). In the subgroup containing upregulated GME3058_g, MGG_05133 was involved in the regulation of conidial differentiation [33], MGG_15508 was associated with pathogenicity [33], while um05801 was reported to be upregulated during the biotrophic growth stage [34]. Combining the evidence of hyphae growth in Figure 2, it is reasonable to deduce that GME3058_g may play similar roles during the gall formation as its homologous genes mentioned above. On the other hand, in the subgroup containing downregulated GME5963_g, um02717 was reported to positively regulate the biosynthesis of ustilagic acid, a toxin that confers *U. maydis* biocontrol activity [35]. Whether GME5963_g also regulates ustilagic acid biosynthesis and whether there is a relationship between this secreted cellobiose glycolipid and gall formation of Jiaobai remains to be elucidated. The expression patterns of *GME3058_g* and *GME5963_g* were further validated by qRT-PCR (Figure 9C).

## 3. Discussion

Jiaobai is a perennial plant with aboveground parts that wither in winter and require several months from sprouting in the spring to the beginning of culm gall expansion. Once a culm gall is formed, it expands rapidly within 1–2 weeks, reaching the size of the commodity period [8,10]. Thus, the initial stage of culm gall formation is a key point to understand the interaction mechanism between *Z. latifolia* and *U. esculenta*. The interaction between the host and pathogen is also very important in the early stage of disease occurrence when the mycelium began to invade the plant cells, such as in rice and blast fungus and maize and *U. maydis* interactions [29,36]. Previous studies have characterized some genes related to stem enlargement in *Z. latifolia* and *U. esculenta* separately [10,37,38], but the analyses were not sufficiently systematic or comprehensive. RNA-seq has been successfully used to study the patterns of global gene expression in both plants and pathogenic fungi during plant–pathogen interactions [28,29,31]. It is difficult to detect the expression of fungal genes in the early stage of infection due to the small proportion of pathogens [29]. *U. esculenta* mycelia were distributed unevenly throughout the culms, and hyphae were abundant in the nodes but rare in the internodes [8]. Our findings showed that the morphology of *U. esculenta* changed dramatically; as the number of hyphae and conidia increased, *U. esculenta* hyphae penetrated the host cells and was mainly distributed in the middle node of the initial stage of the stem gall. Therefore, we selected this tissue for transcriptome sequencing to increase the proportion of pathogens. The results showed that our method could simultaneously detect the expression of a large number of genes in host plants and pathogens during the formation of culm galls in Jiaobai. The proportion of *U. esculenta* genes in the mixed transcriptome of the initial culm gall on day 0 (formation day of Jiaobai) increased compared with that on day −10 (10 days before the expansion of Jiaobai), which was consistent with the increase in *U. esculenta* cells observed in culm gall tissue sections. Concurrently, some genes specific to *Z. latifolia* or *U. esculenta* were expressed at −10 d and 0 d, respectively. These specific genes involved a variety of metabolic pathways and thus might be closely related to the formation of Jiaobai.

The interaction between host plants and pathogens is closely related to plant disease-resistance genes and pathogen genes [32,39,40]. In this study, we observed that a total of 368 plant disease-resistance genes and 71 fungal PHI genes showed significant differences in expression levels. These candidate genes may play important roles in the invasion of *U. esculenta* and the ability of *Z. latifolia* to prevent fungal invasion into cells and stimulate culm gall formation. In pathogenic fungi, chitin synthase not only plays an important role in maintaining cell growth but also has a close relationship with pathogenicity [41,42]. In *Fusarium oxysporum* and *U. maydis*, the mycelia growth and pathogenicity of the chitin synthase-gene-defective mutant were decreased [36,43]. In this study, we found similar results: a large number of hyphae extended into the interior of the host tissue, and the expression of the chitin synthase gene *GME6969_g* in *U. esculenta* was significantly upregulated in the initial stage of culm gall formation. However, we concurrently noticed that some virulence genes associated with strong pathogenicity, such as *GME4426_g*, *GME6815_g*, and *GME6967_g*, were significantly decreased, and a large number of genes involved in the signaling pathway of plant disease resistance, including leucine replication-rich receptor-like kinases, hormone signaling pathways, plant–pathogen interactions and phenylpropanoid biosynthesis pathways, were clearly changed. These results implied that, on the one hand, *U. esculenta* hyphae increased and expanded in the host plant simply to maintain proper pathogenicity without causing fatal disease, and on the other hand, the ability of plants to resist fungal infection increased to maintain the long-term interaction and symbiosis between the fungus and the host during the period of stem-gall expansion.

Jiaobai is produced by the interaction of *Z. latifolia* and *U. esculenta*, and previous studies have shown that plant hormones play an important role in the expansion process of Jiaobai culm gall [7,9,11]. Cytokinin and auxin are two important phytohormones widely reported to induce Jiaobai gall formation [9,10]. Wang et al. (2017) and Li et al. (2021) found that several genes involved in cytokinin and IAA biosynthesis were upregulated at gall initiation [9,10]. However, in our study, only a few genes involves with cytokinin biosynthesis and signaling pathways were found to be upregulated during gall initiation (Figure 6B and Figure S9). IAA content in the initially expanding gall increased significantly, while contents of cytokinin and GA declined, indicating that IAA played a key role in the initial expansion process of Jiaobai. According to the primer sequences, we found that the key gene of IAA biosynthesis in our study (*Zlat_10038559*) is the same gene of *ZlYUCCA* in Wang et al. (2017) [10] and *ZlYUC11* in Li et al. (2021) [9], and the expression of this gene was upregulated in all three experiments at the gall initiation stage. The result further confirms the key roles of IAA and *Zlat_10038559* during the gall formation of Jiaobai. In addition, Li et al. (2021) reported the IAA level was not regulated between −5 d and 5 d of gall formation [9], and the levels of IAA and GA_3_ were much higher than those in our study. We noticed that the days associated with gall formation were defined for sampling in these studies, however, the exact definition of the day before, at or after gall initiation may be different (1 cm in our study and 5 cm in Wang et al. (2017) for 0 d [10], for example). Additionally, as mentioned above, the middle node of the initial stage of the stem gall was sampled for both gene expression and phytohormone assay. Thus, the difference in the sampling time and location may be one of the reasons for the different results among studies. IAA was the first discovered and isolated phytohormone and is the most commonly occurring natural auxin. Interestingly, however, many microorganisms (including bacteria and fungi) have also demonstrated the ability to synthesize IAA, and the role of IAA in plant–microbe interactions has recently received increasing attention [19,44,45]. The function of IAA synthesized by microorganisms or plants is quite different in the initial stage of symptoms. According to some reports, certain plant-growth-promoting rhizobacteria and fungi that produce IAA can induce lateral root formation and root hair development [15,16,46,47]. Phytopathogenic bacteria such as *Agrobacterium tumefaciens* and *Pseudomonas syringae* is synthesize IAA, which is used as a virulence factor to cause tumors and galls [44,46,47,48]. Other reports have suggested that most IAA is contributed by the host plant during the initial critical interaction between tobacco (*Nicotiana tabacum*) and *P. solanaceraum* [15,16]; Reineke et al. (2008) also found that auxin synthase-gene-deletion mutants of smut fungus *U. maydis*-inoculated maize did not affect tumor formation, and considered that fungal IAA production provides a critical contribution to IAA levels in the infected tissue [18]. However, this phenomenon is apparently not important for triggering host tumor formation. Li et al. (2021) found that several genes in IAA biosynthesis and signaling pathways were upregulated during gall formation [9]. To identify if the IAA was produced by *U. esculenta* or its host plant at the initiation stage of gall enlargement, we analyzed the expression of genes encoding key enzymes during IAA synthesis in the two species. We observed that there were no significant differences or even a downregulation of the expression levels of IAA synthesis-related genes of *U. esculenta* at the time of calm gall formation. YUCCA proteins convert indole-3-pyruvate (IPyA) into indole-3-acetic acid (IAA) and are essential for auxin production. They play important roles in plant growth and development. The expression levels of the *YUCCAs* are closely related to the content of IAA, and overexpression of *YUCCA* genes results in elevated auxin levels and auxin-overproduction phenotypes in *Arabidopsis*, rice, potato and cucumber [25,26,27,49]. In addition, Lin et al. [50] found that the expression of *YUCCA* increased significantly during potato tuber formation. In this study, 12 *YUCCA* genes were identified, and further phylogenetic analysis and expression pattern analysis showed that the trend of *ZlYUCCA9* expression and auxin content was consistent during the initial formation of fleshy stems. It was speculated that the auxin triggering calm gall initiation is mainly synthesized by the host plant and not by smut fungi.

**Figure 10:**
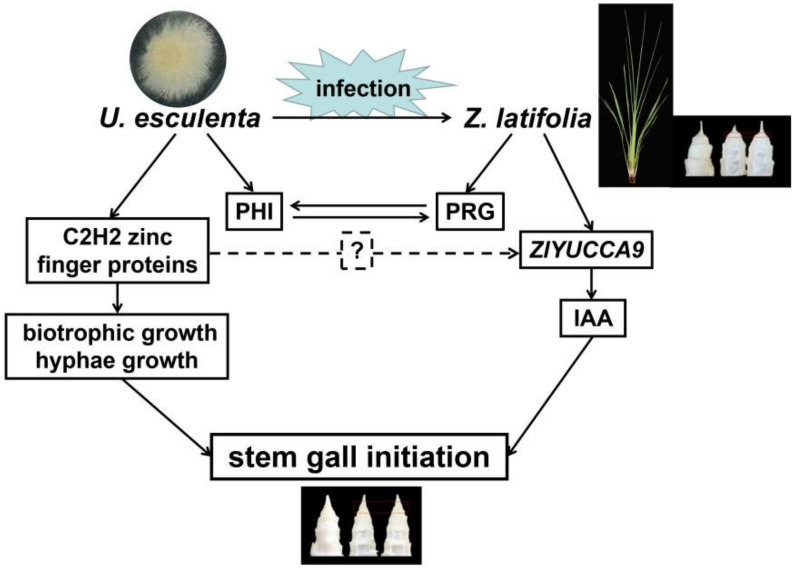
A proposed model of the interaction mechanism between *Z. latifolia* and *U. esculenta* for stem-gall induction deduced from mixed transcriptome analysis. At this stage, the hyphae of *U. esculenta* extend fast and invade into stem apex cells of the host, which may be promoted by the C2H2 zinc finger proteins. The wide distribution of hyphae into the host tissue would facilitate triggering gall enlargement after initiation stage. Fungal pathogen–host interaction genes (PHI) and plant resistant genes (PRG) are reprogrammed to maintain the unique balance between the parasite and the host. IAA stimulating Jiaobao stem-gall initiation is mainly synthesized by *Z. latifolia*, and *ZlYUCCA9* may play a key role in this process. C2H2 zinc finger proteins of *U. esculenta* may also participate into the IAA synthesis of *Z. latifolia*.

There is no doubt that the gall formation of Jiaobai is a result of the interaction of *U. esculenta* and *Z. latifolia*. Therefore, if the IAA inducing gall enlargement initiation is dominantly synthesized by *Z. latifolia*, the role of *U. esculenta* in the gall initiation remains unclear. C2H2 zinc finger protein is one of the largest and most important transcript factor families in fungi and was reported to be associated with a variety of biological process including fungal development, pathogenicity, conidiation, biotrophic growth and so on [33,34,35]. In our study, several *C2H2* genes, closely homologous with these reported genes, were differently expressed during the early gall formation stage. At the same time, significant hyphae growth and invasion into the host cells were also found, indicating similar biological events occurred when the gall started to enlarge. The precondition of fungus triggering gall enlargement is that the fungi should widely distribute into the plant tissue. After that, *U. esculenta* could affect the tissue growth more efficiently by synthesizing phytohormones and other bioactive substances [7,9,17]. To explore whether crosstalk exists between *U. esculenta* and *Z. latifolia* at the early stage of gall formation, the promoter sequence of *ZlYUCCA9* (the −2000 bp upstream from initiation codon) and the protein sequence of GME5963_g were co-analyzed with the JASPAR database (https://jaspar.genereg.net/, accessed on 5 October 2021), and several C2H2 zinc finger protein binding motifs were identified in this region (Table S12). Thus, we could not exclude the possibility of plant–fungus crosstalk in the plant’s auxin biosynthesis pathway (Figure 10).

Taken together, our results indicated that *U. esculenta* grew quickly and invaded host-plant cells at the initial stage of stem-gall formation, accompanied by a large number of gene expression changes in both *Z. latifolia* and *U. esculenta*. Mixed transcriptome analysis helps to elucidate the interactions between host-plant defense and pathogen attack. Auxin plays a key regulatory role in triggering the stem swelling of Jiaobai, and it may be mainly synthesized by *Z. latifolia*, in which *ZlYUCCA9* plays a critical role. GME3058_g and GME5963_g are two important candidate C2H2 zinc finger proteins conducting *U. esculenta* growth and infection in the initial stage of stem-gall formation.

## 4. Materials and Methods

### 4.1. Plant Material and Sample Collection

*Z. latifolia* plants were grown in plastic pots (diameter 53.5 cm and height 37.5 cm) filled with garden soil under natural conditions at the Aquatic Vegetables Experimental Station of Yangzhou University (119°42′ E, 32°24′ N), Yangzhou, Jiangsu Province, China. Seedlings with uniform size at the 4-leaf stage were planted. According to our previous work [51], the stem gall should initiate enlargement about 120 d after planting under certain experimental conditions. Thus, samples were collected at 110 days (noted as −10 d) and 120 days (noted as 0 d) after planting. To increase the proportion of *U. esculenta* in the culm gall, 0.5 cm of the upper part of the gall was removed, while the nodes and the internodes adjacent to them (approximately 0.2 cm) were selected as materials for further analysis (Figure 1C,D). Typical characteristics of the junctions of the leaf sheath and leaves, height, width and color of leaves and stem apex morphology were used to further confirm the development stage of the culm gall. All samples with three biological repeats (approximately 15–20 stems of different plants were collected per replication) were frozen in liquid nitrogen immediately and stored at −80 °C for further experiments.

### 4.2. Distribution of Hyphae in Z. latifolia

The distribution of *U. esculenta* hyphae in *Z. latifolia* was observed using paraffin sectioning and scanning electron microscopy (SEM). According to the size of plant tissue, the whole stem apical tissues of −10 d and five tissue parts (from up to bottom (1–5): stem apex, first node, second node, internode, bottom) of the initial swollen stem (0 d) were used (Figure 1A(a,c)). The paraffin sections were generated as described by Zhang et al. [12], and the morphology of *U. esculenta* in the plant was observed with an Olympus BX-51 microscope (Olympus Optical, Japan). For SEM measurements, the samples were prepared as previously described [52] with slight modifications. The samples were fixed with 2.5% glutaraldehyde and kept overnight at 4 °C. After washing with 0.1 mol/L PBS several times and once with deionized water, they were dehydrated using a graded series of ethanol (30%, 50%, 70%, 80%, 90%, 95%, 100%) and dried in an automated critical point dryer (Leica EM-CPD300, Vienna, Austria). Then, the samples were mounted on aluminum stubs by placing them on double-sided adhesive tape and sputtered with a thin layer of gold using a vacuum sputter coater (Leica-EM SCD500, Germany). SEM images were recorded on a Gemini SEM 300 (Zeiss, German) running at 5.0 kV.

### 4.3. RNA Isolation, mRNA-Seq Library Preparation and Sequencing

Total RNA was extracted from *Z. latifolia* using RNAiso Plus (total RNA-extraction reagent) (Takara, Japan). The concentration of total RNA, Rin value, 28S/18S and fragment size were detected using an Agilent 2100 Bioanalyzer (Agilent RNA 6000 nano Kit), and the RNA purity of the plant and fungal samples was measured with a NanoDrop^TM^. The mRNA was enriched with magnetic beads bearing oligos (dT), double-stranded cDNA was synthesized using the NEBNext^®^ Ultra™ II RNA Library Prep Kit for Illumina, and cDNA was purified with an Agencourt AMPure XP Kit (Beckman, Indianapolis, IN, USA). The constructed library was tested using an Agilent 2100 Bioanalyzer (Agilent, Santa Clara, CA, USA) and a One Step Plus real-time PCR system (ABI, Vernon, CA, USA) and then sequenced using the DNBSEQ^TM^ platform (BGI-Shenzen, China).

### 4.4. Preprocessing of mRNA Sequence Data

The raw RNA-Seq reads were preprocessed with SOAPnuke v1.4.0 [53] (https://github.com/BGI-flexlab/SOAPnuke, accessed on 5 October 2021) to remove reads with adapter contamination, an N base ratio greater than 5%, and low-quality reads. The clean reads were first mapped to the *Z. latifolia* genome (INSDC:ASSH00000000.1) and the *U. esculenta* genome (INSDC: JAAKGJ010000000) [51] using HISAT v2.1.0 (http://www.ccb.jhu.Edu/software/hisat, accessed on 5 October 2021) [54]. After that the Bowtie2 v2.2.5 (http://bowtie-bio.sourceforge.net/bowtie2/index.shtml, accessed on 5 October 2021) was applied to align the clean reads to the gene set [55], then the expression levels of genes and transcripts were quantified using RSEM v1.2.12 (http://deweylab.biostat.wisc.edu/rsem/rsem-calculate-expression.html, accessed on 5 October 2021) [56] and estimated with fragments per kilobase of exon per million mapped reads (FPKM) values. The significantly different DEGs between the two groups were analyzed by DEGseq, where the fold change represented the ratio of the expression level between the two samples, and a fold change ≥ 2 and *p*-value ≤ 0.001 were used as filtering criteria [57].

### 4.5. Functional Annotation of DEGs

Functional annotation of DEGs was accomplished by aligning genes with the NR, Swiss-PROt, GO, COG, KOG and KEGG databases using Blast2GO (http://www.blast2go.org, accessed on 5 October 2021). Plant disease-resistance genes and potential virulence-related proteins were identified by searching against the Plant Resistance Gene Database (PRGdb) and Pathogen–Host Interaction database (PHI-base) using DIAMOND v0.8.31 by BLASTP according to the query coverage and identity with E-values ≤ 1 × 10^−5^.

### 4.6. ESI-HPLC-MS/MS Assay of Endogenous Hormones

The endogenous hormones IAA, ABA, JA, SA, GA_1_, GA_3_, and zeatin were analyzed by HPLC–MS/MS according to the method by Pan et al. (2010) [58]. The 7 analytes were separated using a Poroshell 120 SB-C18 column (2.1 × 150 mm, 2.7 μm) with the mobile phase containing water and methanol in gradient elution mode on an Agilent 1290 high-performance liquid chromatograph (Agilent, Santa Clara, CA, USA). The flow rate was 0.3 mL/min, and the column temperature was 30 °C. Then, a quantitative analysis was performed in multiple-reaction monitoring (MRM) mode with electrospray ionization (ESI) on an AB SCIEX QTRAP 6500+LC-MS/MS system (AB, Fairfield, OH, USA).

### 4.7. Genome-Wide Identification of the IAA Synthesis Genes and C2H2 Zinc Finger Proteins in U. esculenta and Z. latifolia

The protein sequences of TAM, IPDC and IAD in the IPyA pathway, IaaM and iaaH in the IAM pathway, nitrilase in the indole-3-acetonitrile (IAN) pathway, and C2H2 zinc finger proteins of *U. maydis* [18,34], *Neurospora crassa* [20], and *Fusarium proliferatum* [21], and TAA and *YUCCA* in the TAA/YUCCA pathway and IAM pathway of *Arabidopsis* [23,24,26] were used as query sequences to search for the corresponding gene family members in the *U. esculenta* and *Z. latifolia* genomes by BLASTP, and the threshold was set to E < 10^−5^. The protein sequences of the YUCCAs in *Z. latifolia* were aligned with *Arabidopsis* [24], potato [25], and rice [26] using MAFFT [59] and the protein sequences of the C2H2 zinc finger proteins in *U. esculenta* were aligned with *M. oryzae* [33], *U. maydis* [34,35] and *S. cerevisiae* by ClustalW, using default parameters. The phylogenetic analysis was constructed using FastTree [60] with default parameters and displayed using iTol [61]. Furthermore, according to the genomic DNA and CD sequences of *UeC2H2s* and *ZlYUCCAs*, the gene structure was analyzed using the online tool GSDS2.0 (http://gsds.cbi.pku.edu.cn/, accessed on 5 October 2021).

### 4.8. Confirmation of DEGs by qRT-PCR

Several genes associated with the IAA synthesis and two *C2H2* zinc finger protein genes in plants and fungi were selected and confirmed by qRT-PCR. Primers for qRT-PCR were designed using Primer Premier 5.0 and are listed in Table S13 in the Supplementary Materials. PCR amplifications were performed using the Bio-Rad CFX Connect^TM^ Real-Time system (BIO-RAD, Hercules, CA, USA) with a final volume of 20 μL containing 10 μL (2×) iTaq^TM^ Universal SYBR^®^ Green Supermix (BIO-RAD), 1 μL of cDNA, 0.5 μL of forward primer (10 μM), 0.5 μL of reverse primer (10 μM), and 8 μL of sterile water. For each gene, three technical replicates were evaluated. The amplification procedure was as follows: denaturation at 95 °C for 3 min, followed by 39 cycles of 95 °C for 10 s, 56 °C for 30 s, melt curve 60.0 to 95 °C, and increment 0.5 °C for 0.05 s. The transcript level of each gene was normalized against the expression levels of *Z. latifolia* 18S rRNA and *U. esculenta* actin. The 2^−ΔΔCT^ method was used to calculate the relative gene expression levels.

## Figures and Tables

**Table 1:** Statistics for the transcriptome analysis of the Z. *latifolia*-*U. esculenta* interaction in the initial stage of stem-gall formation.

Statistics	*U. esculenta*	*Z. latifolia*
Total clean reads (Gb)	15.25	15.25
Average mapping ratio of genome sequence (%)	4.76	84.03
Average mapping ratio of gene set (%)	3.56	66.82
Total gene number	6330	40,856
Known gene number	6319	37,622
Novel gene number	11	3234
Total novel transcript	1345	36,704
Coding transcript	1013	28,273
Noncoding transcript	332	8431
Novel isoform	1002	24,993
Novel transcript	11	3280

## Supplementary Materials

The following are available online at: https://www.mdpi.com/article/10.3390/ijms222212258/s1.
