# Supplementary material for: Mixed Transcriptome Analysis Revealed the Possible Interaction Mechanisms between Zizania latifolia and Ustilago esculenta Inducing Jiaobai Stem-Gall Formation
Source: Int J Mol Sci. 2021 Nov 12;22(22):12258. doi: 10.3390/ijms222212258 (PMC8618054; doi:10.3390/ijms222212258)
Supplement: Supplementary file 1 [file ijms-22-12258-s001.zip › Supplementary caption.pdf]

Supplementary caption

Supplementary results

The GO and KEGG analysis of DEGs of both and *U. esculenta* and *Z. latifolia*

Supplementary Figures Captions

Figure S1. Pearson correlation coefficient heatmap of all gene expression between the two stages (−10 d before and 0 d) of stem gall formation. A. *U. esculenta.* B. *Z. latifolia*

Figure S2. Functional annotation of DEGs between the two stages (−10 d before and 0 d) of stem gall formation based on the *U. esculenta* genome. A. GO enrichment of DEGs. B. KEGG enrichment of DEGs

Figure S3. Functional annotation of DEGs between the two stages (−10 d before and 0 d) of stem gall formation based on the *U. esculenta* genome. A. GO classification. Each DEG was classified into at least one GO term and grouped into three categories: biological process, cellular component and molecular function. B. Pathway assignment based on KEGG. DEGs were grouped into cellular processes, environmental information processing, genetic information processing, metabolism, and organismal systems

Figure S4. Functional annotation of DEGs between the two stages (−10 d before and 0 d) of stem gall formation based on the *Z. latifolia* genome. A. GO enrichment of DEGs. B. KEGG enrichment of DEGs

Figure S5. Functional annotation of DEGs between the two stages (−10 d before and 0 d) of stem gall formation based on the *Z. latifolia* genome. A. GO classification of DEGs. B. Pathway assignment based on KEGG of DEGs

Figure S6. DEGs of PR genes in the initial stage of stem gall formation

Figure S7. The complete protein sequences of 11 DEGs C2H2 zinc finger proteins were aligned by ClustalW

Figure S8. Structural analysis of the C2H2 zinc finger proteins family.

Supplementary Tables Captions

Table S1. Total mapping to the genome and gene set of *Z. latifolia-U. esculenta* interaction in the initial stage of stem gall formation

Table S2. GO enrichment analysis of DEGs of *U. esculenta* in the initial stage of stem gall formation

Table S3. KEGG enrichment analysis of DEGs of *U. esculenta* in the initial stage of stem gall formation

Table S4. List of DEGs of *U. esculenta* involved in KEGG pathways in the initial stage of stem gall formation

Table S5. GO enrichment analysis of DEGs of *Z. latifoli*a in the initial stage of stem gall formation

Table S6. KEGG enrichment analysis of DEGs of *Z. latifolia* in the initial stage of stem gall formation

Table S7. DEGs related to plant hormone metabolism and signaling of *Z. latifolia* in the initial stage of stem gall formation

Table S8. Genes involved in IAA biosynthesis in *U. esculenta* and *Z. latifolia*

Table S9. Conserved motifs in *Z. latifolia* YUCCA proteins

Table S10. The expression profiles of genes involved in IAA biosynthesis in *U. esculenta* and *Z. latifolia*

Table S11. C2H2 zinc finger proteins of *U. esculenta*

Table S12. Prediction of C2H2 zinc finger binding sites in the promoter region of *ZlYUCCA9*

Table S13. Primer sequences for qRT-PCR
