# Supplementary material for: Mixed Transcriptome Analysis Revealed the Possible Interaction Mechanisms between Zizania latifolia and Ustilago esculenta Inducing Jiaobai Stem-Gall Formation
Source: Int J Mol Sci. 2021 Nov 12;22(22):12258. doi: 10.3390/ijms222212258 (PMC8618054; doi:10.3390/ijms222212258)
Supplement: Supplementary file 1 [file ijms-22-12258-s001.zip › Supplementary Materials Figures/Supplementary materials Figure Captions.pdf]

Supplementary materials

Supplementary Figures Captions

Figure S1. Pearson correlation coefficient heatmap of all gene expression between the two stages (−10 d before and 0 d) of stem gall formation. A. *U. esculenta*. B. *Z. latifolia*

Figure S2. Functional annotation of DEGs between the two stages (−10 d before and 0 d) of stem gall formation based on the *U. esculenta* genome. A. GO enrichment of DEGs. B. KEGG enrichment of DEGs

Figure S3. Functional annotation of DEGs between the two stages (−10 d before and 0 d) of stem gall formation based on the *U. esculenta* genome. A. GO classification. Each DEG was classified into at least one GO term and grouped into three categories: biological process, cellular component and molecular function. B. Pathway assignment based on KEGG. DEGs were grouped into cellular processes, environmental information processing, genetic information processing, metabolism, and organismal systems

Figure S4. Functional annotation of DEGs between the two stages (−10 d before and 0 d) of stem gall formation based on the *Z. latifolia* genome. A. GO enrichment of DEGs. B. KEGG enrichment of DEGs

Figure S5. Functional annotation of DEGs between the two stages (−10 d before and 0 d) of stem gall formation based on the *Z. latifolia* genome. A. GO classification of DEGs. B. Pathway assignment based on KEGG of DEGs

Figure S6. DEGs of plant disease resistance genes in the initial stage of stem gall formation

Figure S7. The complete protein sequences of 11 DEGs C2H2 zinc finger proteins were aligned by ClustalW

Figure S8. Structural analysis of the C2H2 zinc finger proteins family.

Figure S9. Validation of the RNA-Seq data in the cytokinin biosynthesis and signal pathway using qRT-PCR at the initial stage of stem gall formation.
