# Supplementary material for: Mixed Transcriptome Analysis Revealed the Possible Interaction Mechanisms between Zizania latifolia and Ustilago esculenta Inducing Jiaobai Stem-Gall Formation
Source: Int J Mol Sci. 2021 Nov 12;22(22):12258. doi: 10.3390/ijms222212258 (PMC8618054; doi:10.3390/ijms222212258)
Supplement: Supplementary file 1 [file ijms-22-12258-s001.zip › Supplementary Materials tables/Supplementary materials Table S1.pdf]

Supplementary Table

Table S1 Total mapping to the genome and gene set of *Z. latifolia-U. esculenta* interaction in the initial stage of stem gall formation

Species	Total Mapping of genome (%)			Total Mapping of gene set (%)		
	- 10 d	0 d	Average value	- 10 d	0 d	Average value
Z.latlfolia	84.9	83.1	84.03	67.5	66.1	66.82
U. esculenta	3.87	5.64	4.76	2.88	4.24	3.56
