# Supplementary material for: Mixed Transcriptome Analysis Revealed the Possible Interaction Mechanisms between Zizania latifolia and Ustilago esculenta Inducing Jiaobai Stem-Gall Formation
Source: Int J Mol Sci. 2021 Nov 12;22(22):12258. doi: 10.3390/ijms222212258 (PMC8618054; doi:10.3390/ijms222212258)
Supplement: Supplementary file 1 [file ijms-22-12258-s001.zip › Supplementary results.pdf]

*Supplementary results*

# The GO and KEGG analysis of DEGs of both *Z. latifolia* and *U. esculenta*

*1 Functional annotation and classification based on the U. esculenta genome*

1.1. GO analysis of DEGs of *U. esculenta*

According to the analysis of gene function, 129 DEGs between the -10 d and 0 d lines were involved in the enrichment analysis and the functional classification of Gene Ontology (GO). Significant enrichment analysis of GO was conducted for DEGs between -10 d and 0 d of the stem apical meristem using the Phyper function in R software, and the P-value was calculated and corrected by FDR. There were 7 GO terms with more than 10 DEGs, which were catalytic activity (GO:0003824), membrane (GO:0016020), integral component of membrane (GO:0016021), intrinsic component of membrane (GO:0031224), membrane part (GO:0044425), oxidoreductase activity (GO:0016491), and transmembrane transport (GO:0055085) and contained 67, 49, 48, 48, 48, 23 and 12 DEGs, respectively (Figure S2A, Table S2). Generally, functions with a Q-value ≤0.05 were regarded as significant GO enrichment, and two GO terms for integral component of membrane, and intrinsic component of membrane were obtained. To gain more insights into the functions of DEGs, 129 DEGs were classified into three categories: biological process, cellular component and molecular function (Figure S3A). In the biological process category, the DEGs were mainly involved in metabolic process, cellular process and localization, with 40, 34 and 16 DEGs, respectively. A large number of DEGs in cellular component were mainly distributed in the membrane, membrane part, cell and organelle, with 49, 48, 38 and 26 DEGs, respectively, followed by organelle part, macromolecular complex, cell part, membrane-enclosed lumen and extracellular region. Within the molecular function category, most of the DEGs were found to be involved in binding and catalytic activity, representing in the top twoin all GO categories. In addition, the proportion of DEGs was higher in the cellular component and molecular function categories, response to extracellular region, signal transducer activity, transcription regulator activity in the 0 d lines than in the control. In contrast, the DEGs involved in detoxification, signaling, antioxidant activity and molecular function regulator were expressed at lower levels at 0 d than at -10 d (Figure S3A).

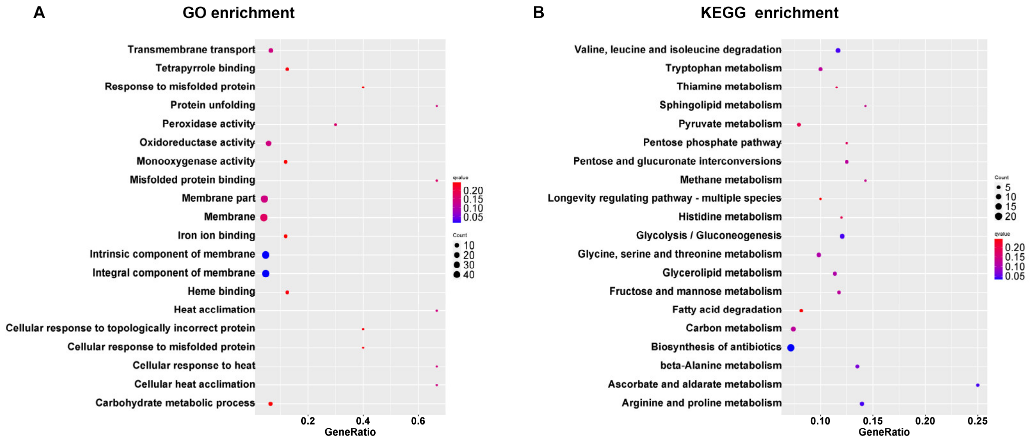


**Figure S2.** Functional annotation of DEGs between the two stages (−10 d before and 0 d) of stem gall formation based on the *U. esculenta* genome. A. Gene ontology (GO) enrichment of DEGs. B. KEGG enrichment of DEGs.

1.2. KEGG pathway analysis of DEGs in *U. esculenta*

The Phyper function in R software was used to determine significantly enriched KEGG pathways with a corrected Q-value ≤0.05 as the screening threshold, and 5 KEGG pathways

were found to be significantly enriched in 20 DEGs involved in biosynthesis of antibiotics (ko01130), 7 DEGs in glycolysis/gluconeogenesis (ko00010), 4 DEGs in ascorbate and aldarate metabolism (ko00053), 7 DEGs in valine, leucine and isoleucine degradation (ko00280), and 6 DEGs in arginine and proline metabolism (ko00330)(Figure S2B, Table S3). According to KEGG annotation analysis and official classification, DEGs between the -10 d and 0 d samples were classified into 6 branches: cellular processes, environmental information processing, genetic information processing, human disease, metabolism and organismal systems. A total of 100 DEGs were annotated in 19 KEGG pathways and mainly focused on metabolism concentrated in global and overview maps, carbohydrate metabolism and amino acid metabolism with 54, 32 and 20 DEGs, respectively (Figure S3B). Further analysis showed that the expression of 8 DEGs, *GME2906_g* and *GME5929_g* in membrane transport, *GME2286_g* in endocrine and metabolic diseases, GME6512_g in biosynthesis of other secondary metabolites and *GME1592_g*, *GME5593_g*, *GME6081_g*, and *GME6343_g* in energy metabolism were downregulated in the initial stage of culm gall formation. We also found that the expression of *GME6969_g*-encoding enzymes responsible for the synthesis of chitin, which is related to the maintenance of cell wall integrity, was significantly increased during gall enlargement of *Z. latifolia* (Table S4).

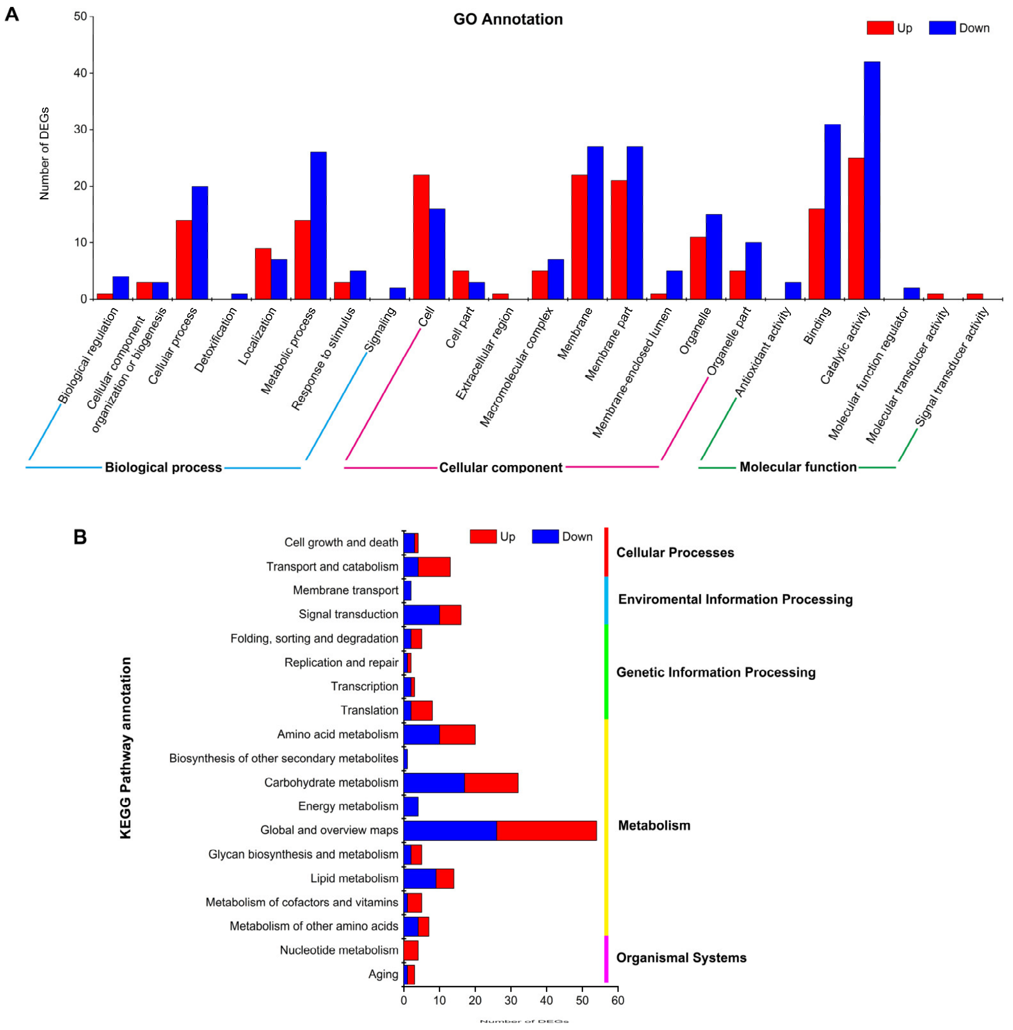


**Figure S3.** Functional annotation of DEGs between the two stages (−10 d before and 0 d) of stem gall formation based on the U.esculenta genome. A. GO classification. Each DEG was classified into at least one GO term, and grouped into three categories: biological process, cellular component and molecular function. B. Pathway assignment based on KEGG. DEGs

were grouped into cellular processes; environmental information processing; genetic information processing; metabolism; organismal systems.

*2. Functional annotation and classification based on the Z. latifolia genome*

2.1. GO analysis of DEGs of *Z. latifolia*

A total of 116 GO terms of *Z. latifolia* DEGs were significantly enriched in the initial stage of stem gall formation (Table. S5). The enrichment bubble diagram shows the 20 GO terms with the minimum Q-value, and the top 10 most significantly enriched terms in DEGs were transcription, DNA-templated (GO:0006351), RNA biosynthetic process (GO:0032774), nucleic acid-templated transcription (GO:0097659), tetrapyrrole binding (GO:0046906), heme binding (GO:0020037), nucleobase-containing compound biosynthetic process (GO:0034654), oxidoreductase activity (GO:0016491), organic cyclic compound biosynthetic process (GO:1901362), regulation of hormone levels (GO:0010817), and extracellular region (GO:0005576) (Figure S4A). To further understand the function of DEGs, we compared them with the whole expression background to obtain GO classification information with potential enrichment functions in DEGs, and a total of 2774 DEGs were involved in 48 functional categories. The top five categories were related to binding, cell, catalytic activity, cellular process, and metabolic process, with 1471, 1422, 1345, 1,266 and 1,196 genes, and there were 695 and 776, 879 and 543, 591 and 754, 631 and 635, and 389 and 484 upregulated and downregulated genes, respectively (Figure S5A).

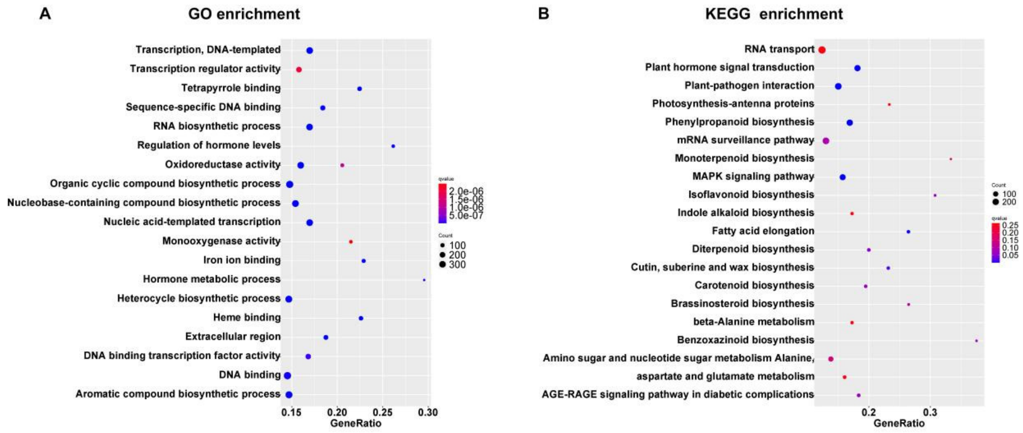


**Figure S4.** Functional annotation of DEGs between the two stages (−10 d before and 0 d) **of** stem gall formation based on the *Z. latifolia* genome. A. GO enrichment of DEGs. B. KEGG enrichment of DEGs.

2.2. KEGG pathway analysis of DEGs in *Z. latifolia*

During stem gall formation, the top 20 pathways with a high reliability of DEG enrichment in the KEGG pathway classification groups were compared and analyzed, and the results showed that the pathways enriched with more than 100 DEGs were RNA transport (ko03013), mRNA surveillance pathway (ko03015), plant-pathogen interaction (ko04626), phenylpropanoid biosynthesis (ko00940), plant hormone signal transduction (ko04075), MAPK signaling pathway - plant (ko040160), endocytosis (ko04144), and amino sugar and nucleotide sugar metabolism (ko00520) (Figure S4B). A total of 6 metabolic pathways with significantly enriched DEGs were screened, followed by plant hormone signal transduction, phenylpropanoid biosynthesis, plant-pathogen interaction, MAPK signaling pathway - plant, fatty acid elongation (ko00062), cutin, suberine and wax biosynthesis (ko00073) (Table. S6). In particular, 185 DEGs were enriched in the plant hormone signal transduction pathway, including 106 upregulated and 79 downregulated genes, which may be related to stem gall formation in *Z. latifolia.*

Furthermore, 2074 DEGs were involved in cellular processes, environmental information processing, genetic information processing, human diseases, metabolism, and organismal systems based on the *Z. latifolia* genome (Figure S5B). Of these, the largest number of categories were metabolites, which were mainly involved in global and overview maps (958 DEGs), carbohydrate metabolism (355 DEGs), biosynthesis of other secondary metabolites (258 DEGs), lipid metabolism (168 DEGs) and amino acid metabolism (157 DEGs). The second was genetic information processing, which involved translation (376 DEGs), transcription (130 DEGs), replication (43 DEGs) and repair and folding, sorting and degradation (184 DEGs). Among them, the numbers for cellular processes, environmental information processing, human diseases and organismal systems was relatively small at 159, 378, 29 and 267 DEGs, respectively, which were mainly involved in signal transduction (352 DEGs), environmental adaptation (267 DEGs) and transport and catabolism (159 DEGs).

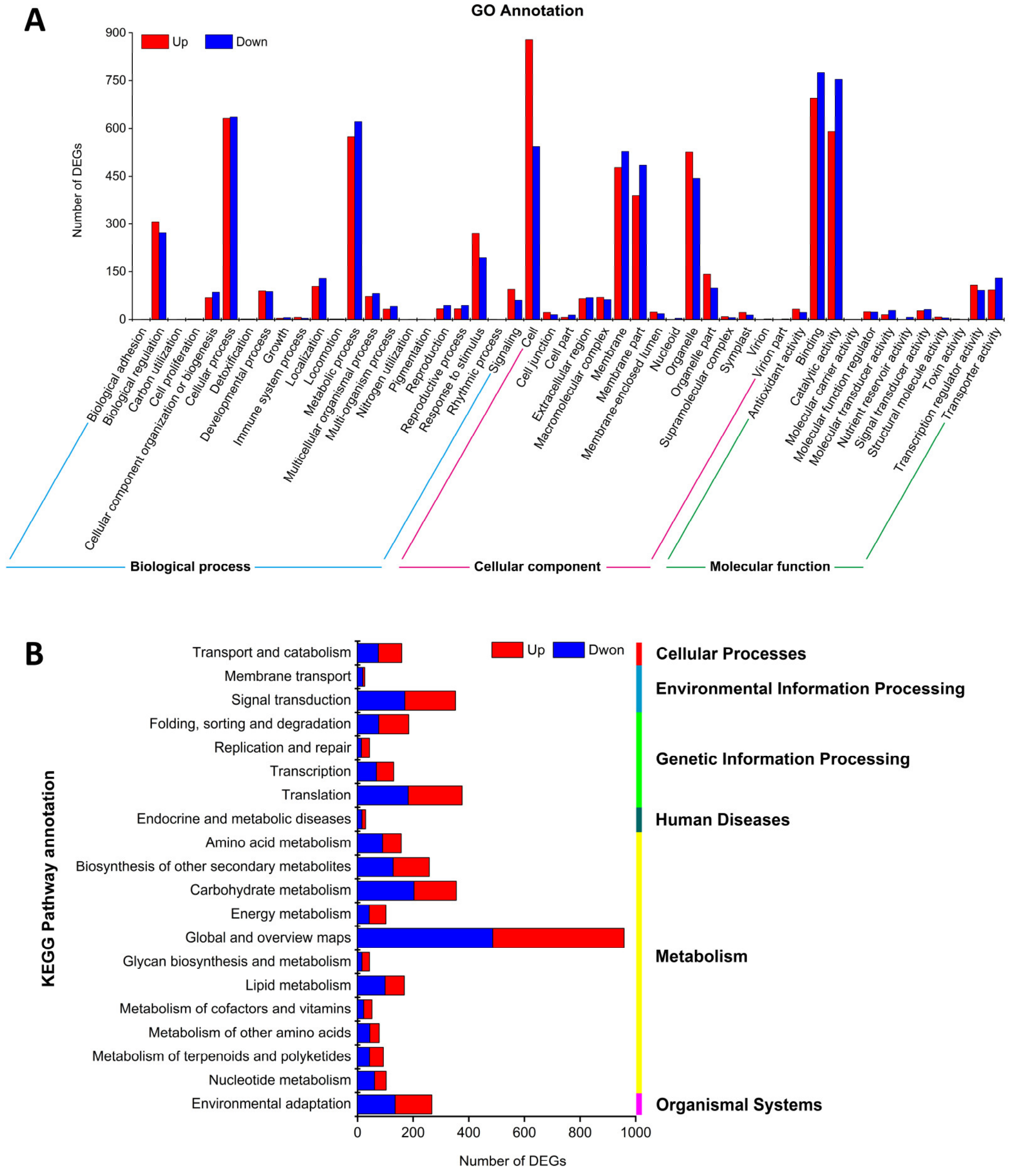


**Figure S5.** Functional annotation of DEGs between the two stages (−10d before and 0 d) of stem gall formation based on the Z. latifolia genome. A. GO classification of DEGs. B. Pathway assignment based on KEGG of DEGs.
