# Supplementary figures and images for: Mixed Transcriptome Analysis Revealed the Possible Interaction Mechanisms between Zizania latifolia and Ustilago esculenta Inducing Jiaobai Stem-Gall Formation
Source: Int J Mol Sci. 2021 Nov 12;22(22):12258. doi: 10.3390/ijms222212258 (PMC8618054; doi:10.3390/ijms222212258)

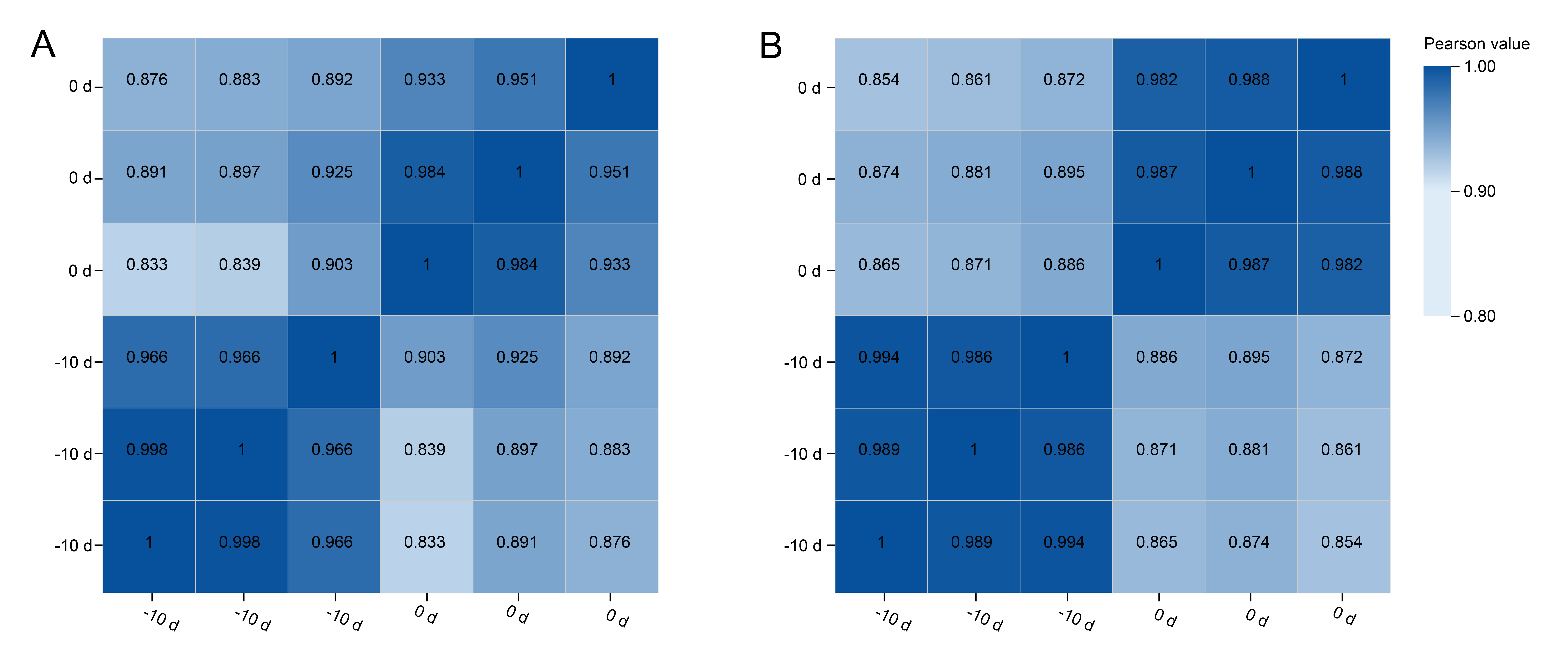

Supplement: Supplementary file 1 [file ijms-22-12258-s001.zip › Supplementary Materials Figures/Fig. S1.jpg]

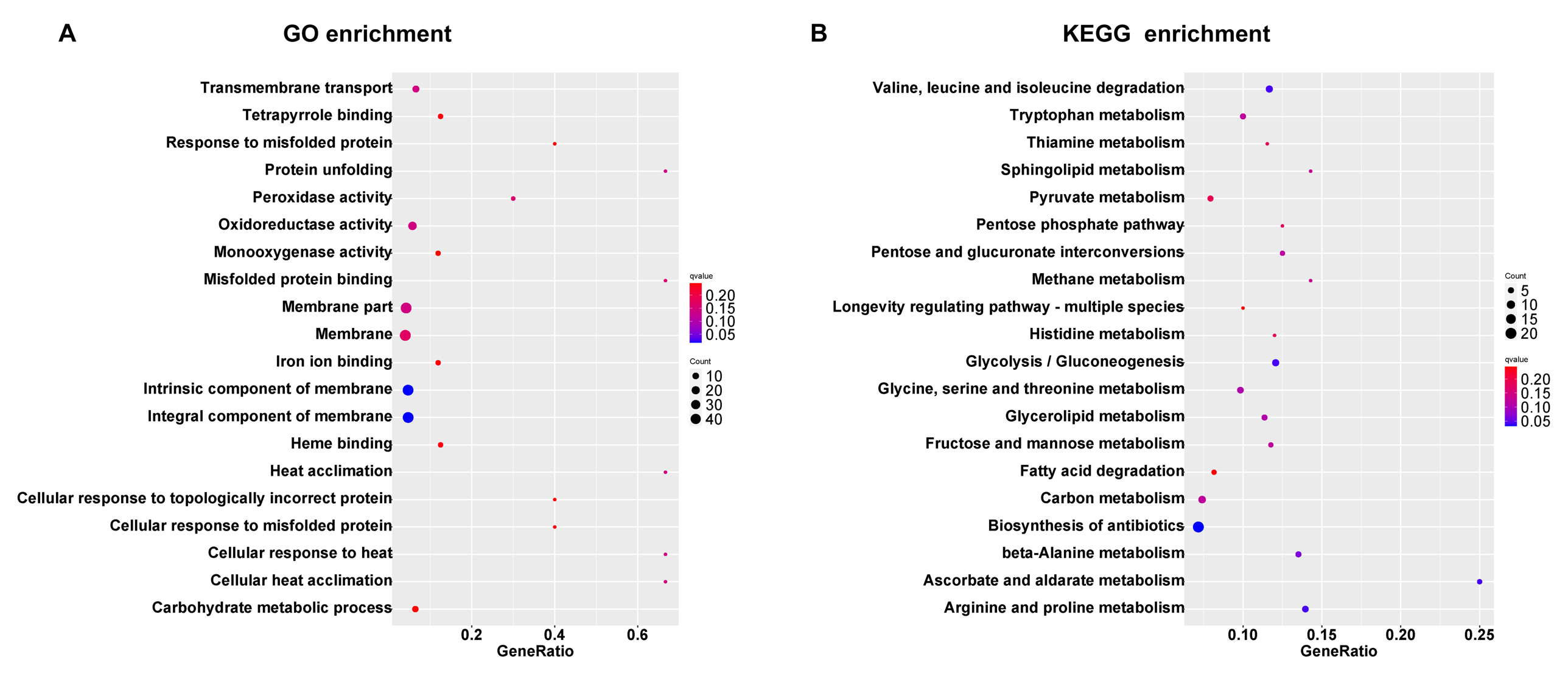

Supplement: Supplementary file 1 [file ijms-22-12258-s001.zip › Supplementary Materials Figures/Fig. S2.jpg]

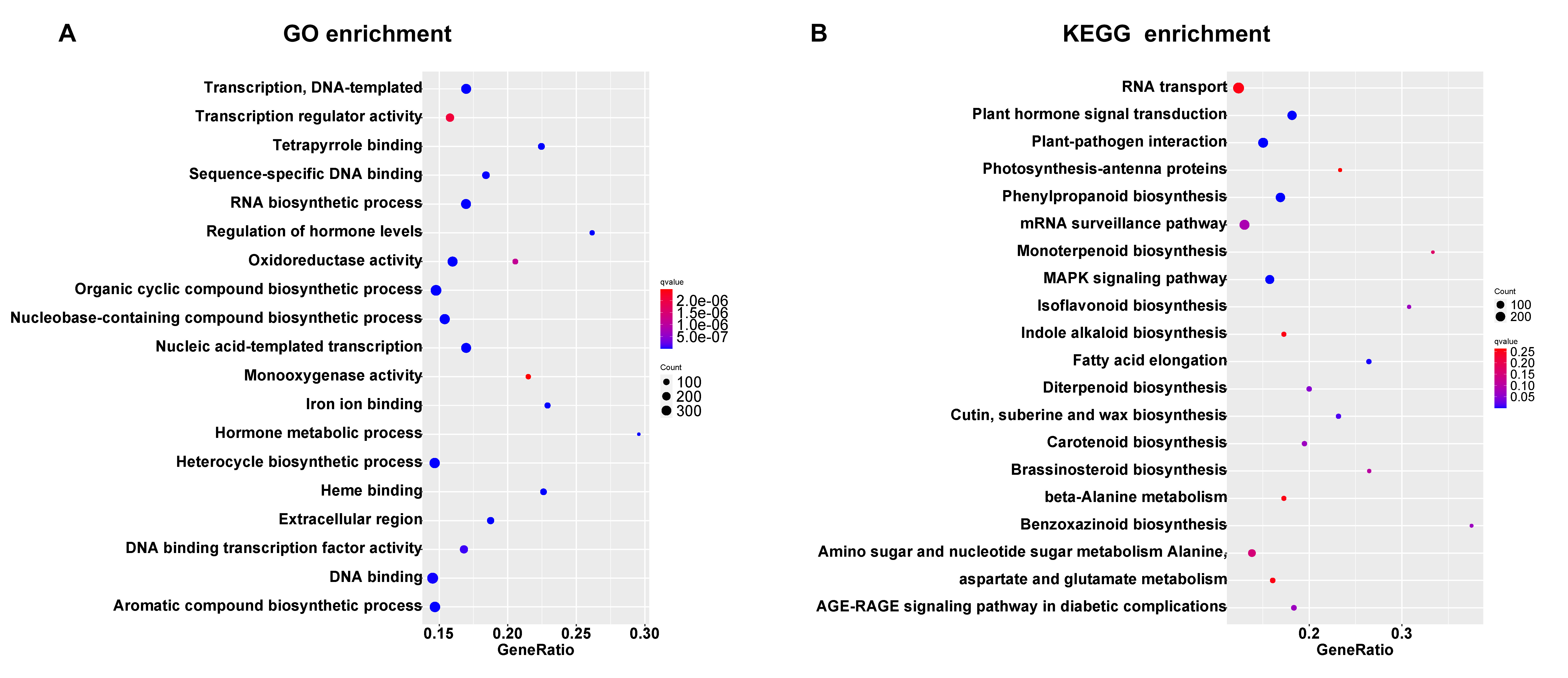

Supplement: Supplementary file 1 [file ijms-22-12258-s001.zip › Supplementary Materials Figures/Fig. S4.jpg]

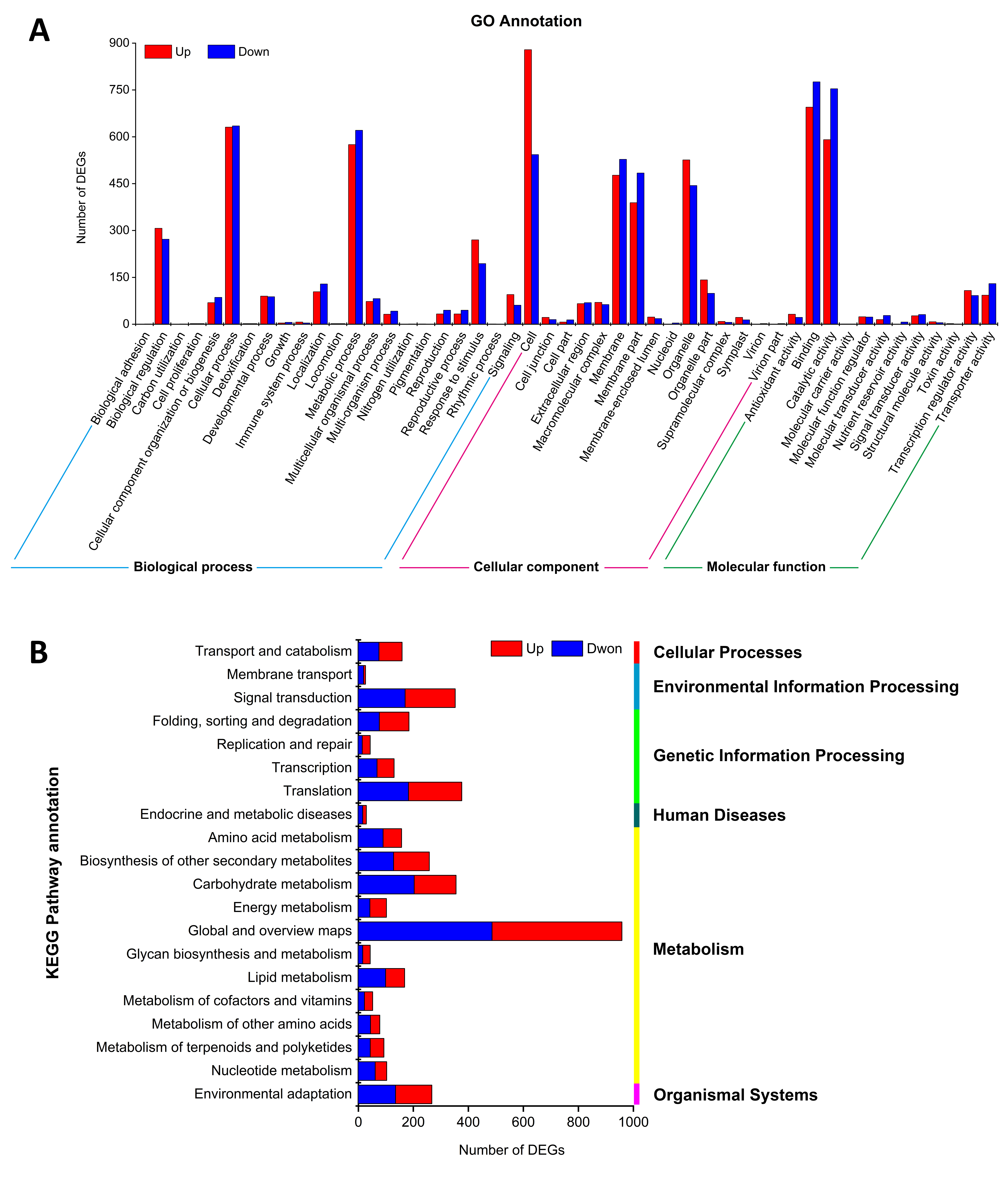

Supplement: Supplementary file 1 [file ijms-22-12258-s001.zip › Supplementary Materials Figures/Fig. S5.jpg]

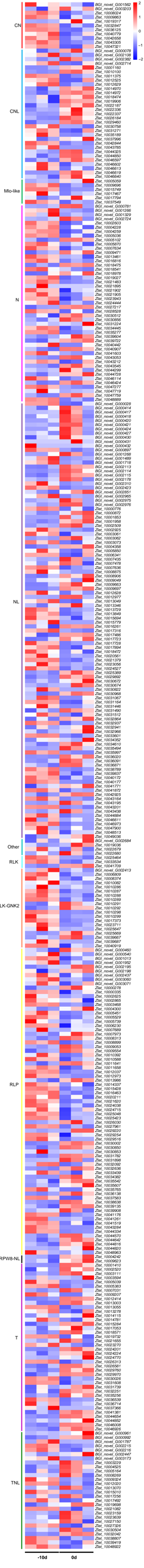

Supplement: Supplementary file 1 [file ijms-22-12258-s001.zip › Supplementary Materials Figures/Fig. S6.pdf]

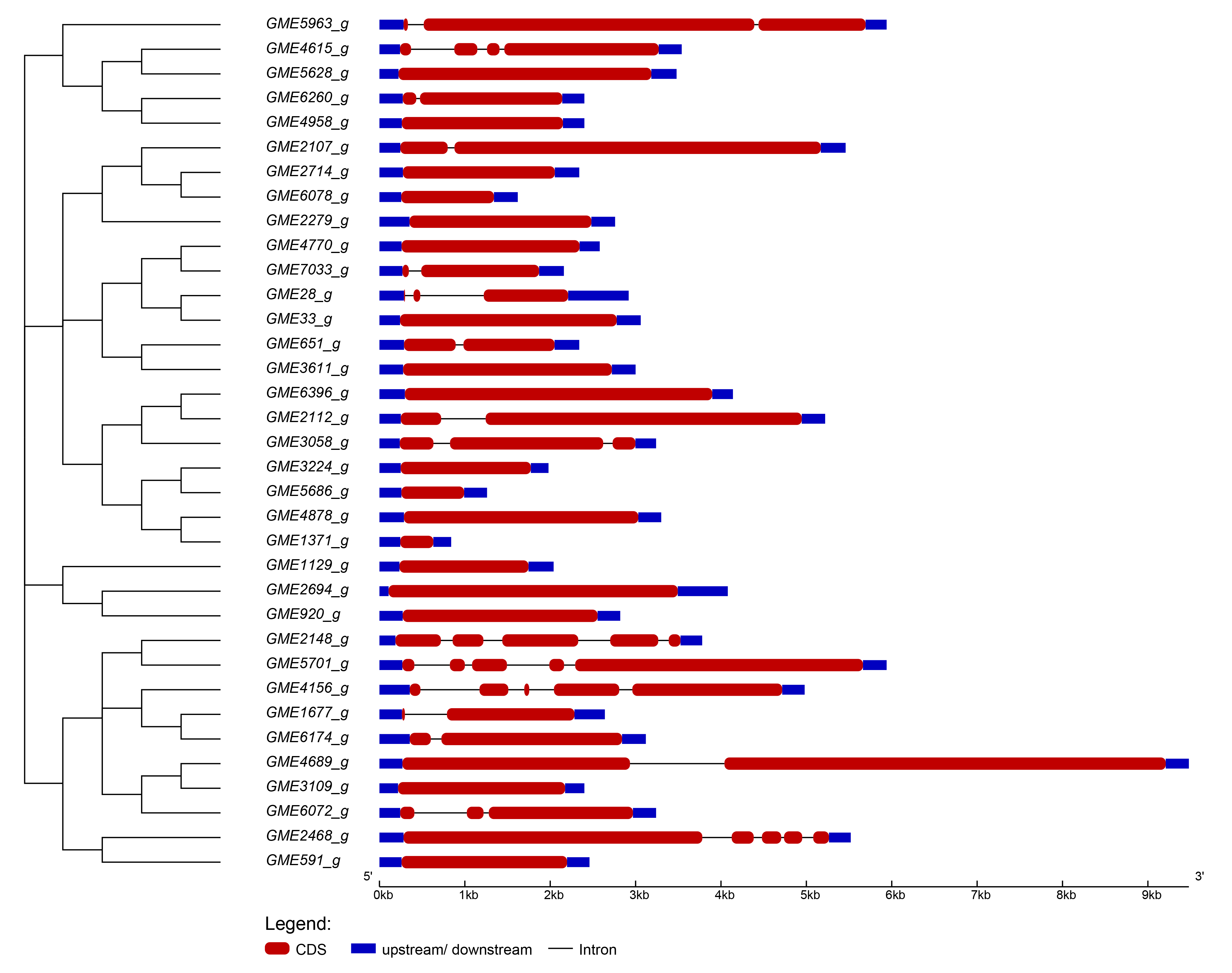

Supplement: Supplementary file 1 [file ijms-22-12258-s001.zip › Supplementary Materials Figures/Fig. S8.jpg]

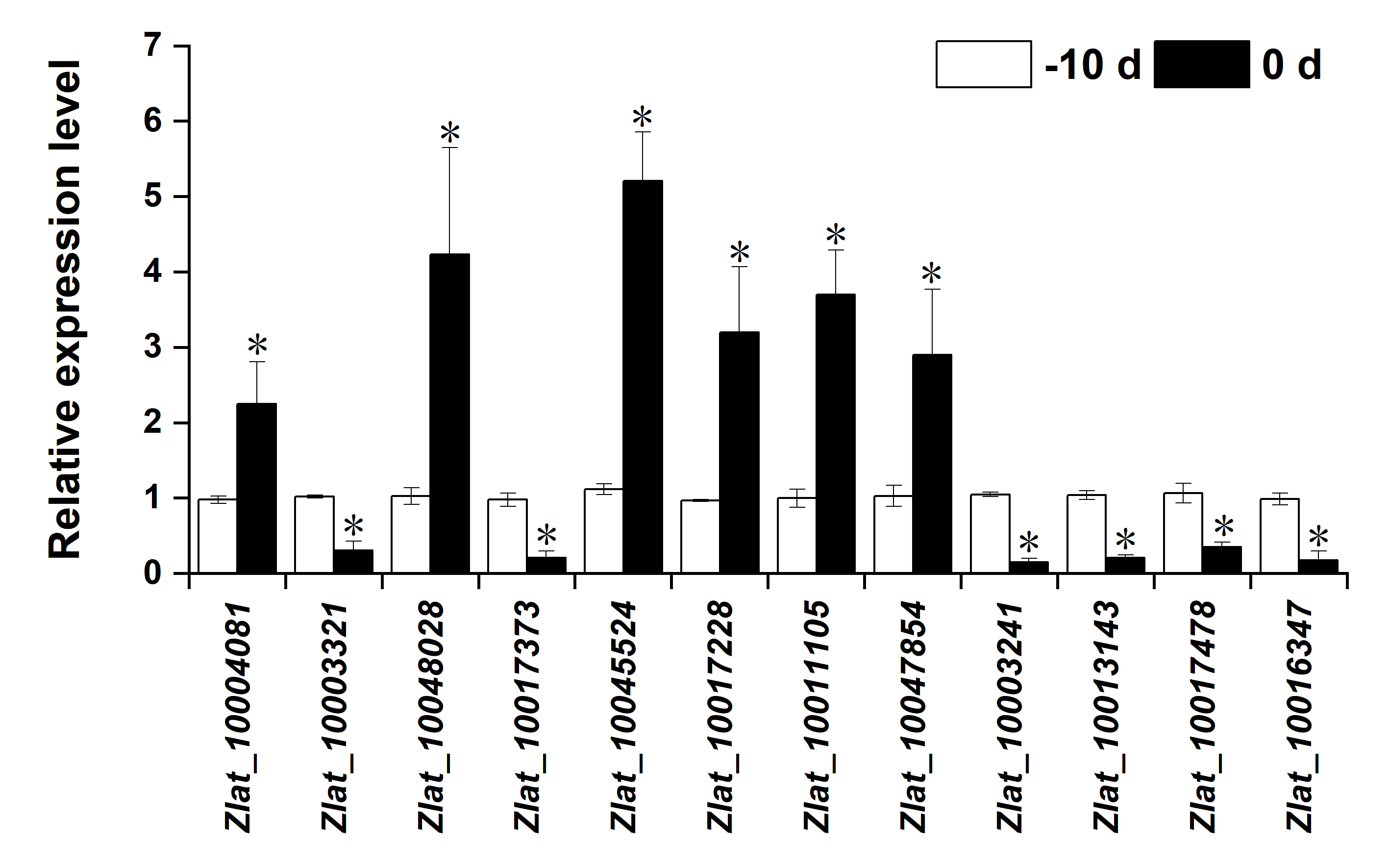

Supplement: Supplementary file 1 [file ijms-22-12258-s001.zip › Supplementary Materials Figures/Fig. S9.jpg]
