# Supplementary material for: Mixed Transcriptome Analysis Revealed the Possible Interaction Mechanisms between Zizania latifolia and Ustilago esculenta Inducing Jiaobai Stem-Gall Formation
Source: Int J Mol Sci. 2021 Nov 12;22(22):12258. doi: 10.3390/ijms222212258 (PMC8618054; doi:10.3390/ijms222212258)
Supplement: Supplementary file 1 [file Fig. S7.pdf › Supplementary Materials Figures/Fig. S7.pdf]

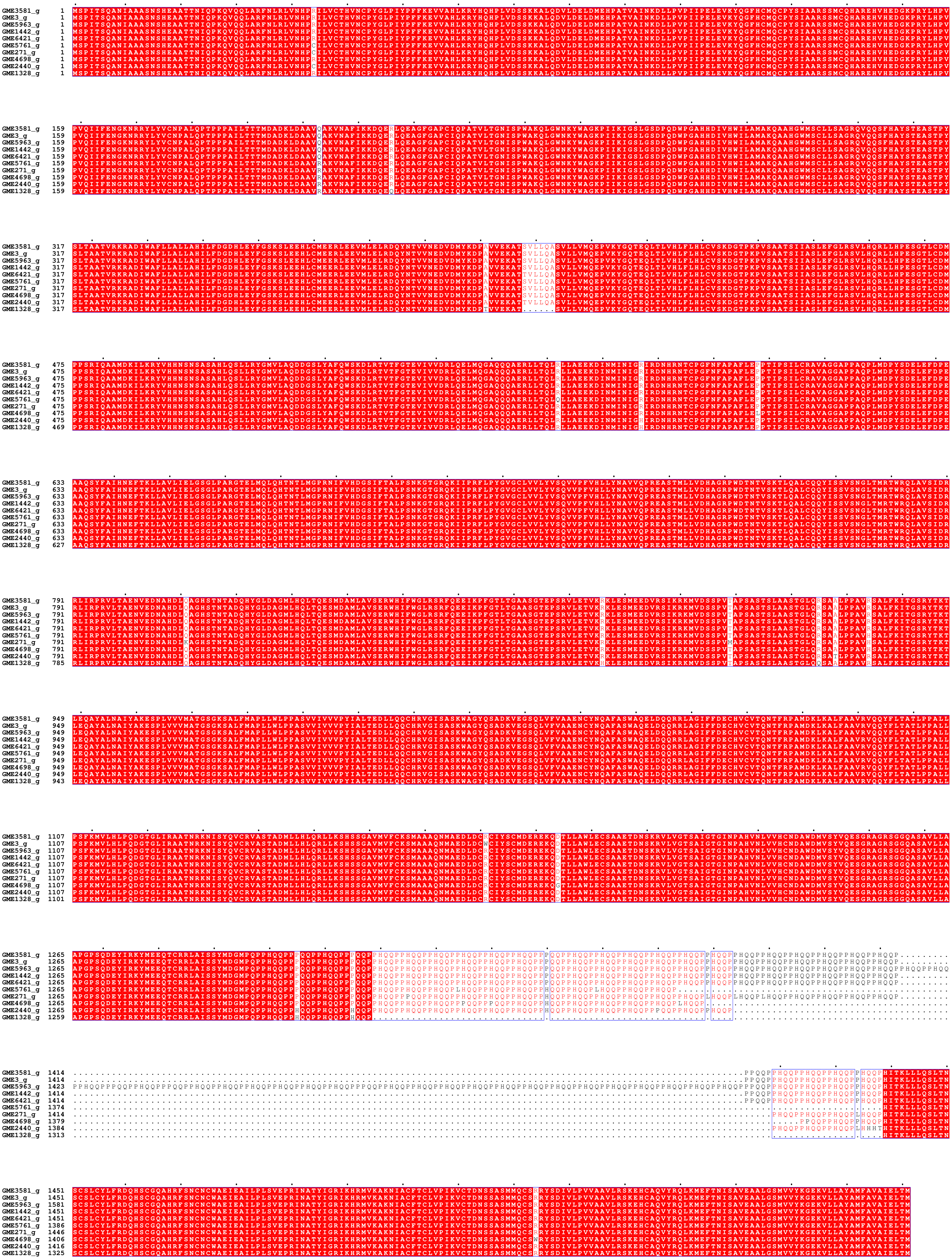
GME3581_g
GME3_g
GME5963_g
GME1442_g
GME6421_g
GME5761_g
GME271_g
GME4698_g
GME2440_g
GME1328_g
